# Control of heart rate through guided high-rate breathing

**DOI:** 10.1038/s41598-018-38058-5

**Published:** 2019-02-07

**Authors:** Sean Perry, Natasha A. Khovanova, Igor A. Khovanov

**Affiliations:** School of Engineering, University of Warwick, Coventry, CV4 7AL United Kingdom

## Abstract

Understanding the complex dynamics of cardio-respiratory coupling sheds light on the underlying mechanisms governing the communication between these two physiological systems. Previous research has predominantly considered the coupling at respiratory rates slower than the heart rate and shown that respiratory oscillations lead to modulation and/or synchronization of the heart rate. Whereas the mechanisms of cardio-respiratory communication are still under discussion, peripheral nervous regulation is considered to be the predominant factor. This work offers a novel experimental design and applies the concept of instantaneous phase to detect cardio-respiratory entrainment at elevated respiration rates, close to the resting heart rate. If such 1:1 entrainment exists, it would suggest direct neuronal communication between the respiration and heart centres in the brain. We have observed 1:1 entrainment in all volunteers, with consistently longer synchronization episodes seen in physically fitter people, and demonstrated that cardio-respiratory synchronization at both low and high respiration rates is associated with a common underlying communication mechanism.

## Introduction

The interaction between the cardiac and respiratory systems is important for effective and efficient gas exchange^1^. It involves the autonomous nervous system and occurs via a variety of mechanical and neuronal regulatory mechanisms^2^ which are not fully understood. For example, the role of the central neuronal networks and mechanically induced reflexes in the modulation of the heart rate by respiration is a subject of scientific discussion^3,4^. This modulation is known as respiratory sinus arrhythmia (RSA)^5^, which manifests itself through the number of heart beats per breath changing according to the respiration cycle, with the heart rate increasing during inspiration and decreasing during expiration. Understanding the origin of the cardiac and respiratory rhythms and their coupling, as well as the role of mechanical and neuronal regulatory mechanisms, is essential for a better characterization of RSA and other interactive effects reported in the literature^6–9^.

It is known that cardiac and respiratory systems are characterized by their own rhythms, which are generated by different neural centres located in the medulla oblongata^2^. In turn, interactions between the centres via coupling and feedback loops affect the individual cardio and respiratory rhythms; such interactions can be characterized through the analysis of rhythm alternations^10,11^. Cardio-respiratory interaction stronger than that during RSA is observed during cardio-respiratory synchronization (CRS), with a specific number *m* heart beats per *n* respiration cycles (locking ratio *n*:*m*)^6–8^. During CRS the heartbeats are only observed for specific phases of the respiratory cycle. CRS is more pronounced in athletes and occurs irregularly^10,12^. The irregularity is often explained by the presence of noise, i.e. many factors other than respiration influencing cardio-respiratory interactions. Recent developments have demonstrated the usefulness of a phase description^13^ of both RSA and CRS. According to the phase description, RSA can be represented as a continuous increase and decrease of the heart rate with respect to the phase of respiratory cycle^10^. CRS assumes constant phase difference for different locking ratios *n*:*m* between the heart (*m*) and respiratory (*n*) rates^7,10^; CRS is typically considered for respiratory rates slower than the heart rate, i.e. for *n* < *m*.

Several experimental observations, for example by Koephen^14^, have suggested that cardio and respiratory systems are coupled peripherally as well as centrally. Both RSA and CRS are considered the product of peripheral coupling^15,16^; such peripheral links are characterized by the time delays between the action of one system and the reaction of another, with the time scales of the delays being smaller than the slow changing respiration. Furthermore, when the two rhythms are similar in pace, central neural coupling might also occur under certain conditions. Koephen postulated that for such central coupling to exist there must be a common central neuronal source by which the two rhythms are coordinated^14^. The central coupling has been considered in a series of experiments by Pokrovskii (see^17^ and references therein). They proposed^18^ the existence of a cardiac rhythm generator in the central nervous system and a direct connection between this centre and the respiratory centre, both located in the medulla oblongata. As a manifestation of this direct link, the phenomenon of CRS with locking ratio 1:1 was discussed^19–21^ suggesting that cardio-respiratory entrainment can be achieved by practising breathing at a rate greater than the resting heart rate (RHR)^21^, which is the heart rate when breathing rate is normal. A flashing light and auditory signal were used as a guide for the participants to keep their respiration rate above the RHR; participants were typically in a standing position. By changing the flash rate in 5% increments with respect to the RHR, a region of respiratory frequency with CRS was identified. The existence of synchronization was verified by a visual inspection of the recordings of each heartbeat and breath wave. Measurements were carried out for a large population. However in this research^19–21^ no significance is placed upon length of synchronization episodes, nor are any statistical measures applied to verify the strength of the interconnection. The methodology used by the group^19–21^ to identify CRS was described as purely visual. The lack of rigorous data analysis does not allow for identification of coincidental equivalent rates versus true entrainment, as discussed previously^6^.

The aim of this work is to investigate whether 1:1 phase-locking CRS can be observed for extended periods of time, and to describe the phenomenon in appropriate mathematical terms by designing an experiment to guide high-rate respiration from a basic rhythm corresponding to a slightly lower rate than the RHR, to higher rhythms, above the RHR. If such 1:1 entrainment exists, it would suggest that the respiratory centre in the medulla could become the dominant pacemaker controlling the heart rate and such an observation would suggest *direct neuronal communication*. Inspiration for instigating CRS is taken from Pokrovskii’s work^19–21^, however a novel experimental design is proposed and a rigorous methodology for data analysis has been developed. To reduce the influence of the visual and auditory neural centres and mechanical cardio-respiratory coupling the light flash is replaced by a visual pattern and the measurements are taken with subjects in a lying position. Furthermore, longer intervals of high-rate breathing was used that, in a combination with the developed methodology, allow robust identification of entrainment between the cardiac and respiratory systems by avoiding solely coincidental rate equivalency^6^. We also aim to consider the adaptation of heart dynamics to a step change in respiration rhythm.

## Results

### Measurements

**Figure 1:**
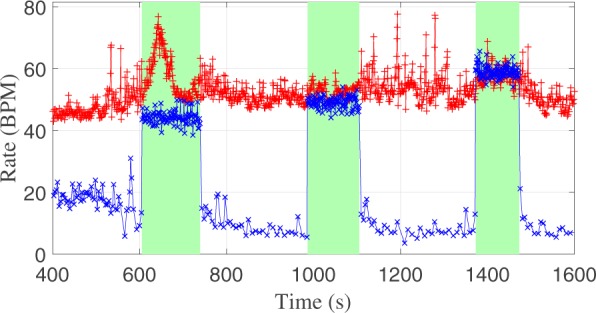
Instantaneous breathing rate (blue) and heart rate (red) are shown from data for volunteer 11. Regions of guided breathing are shaded. Markers ‘x’ and ‘+’ correspond to maxima in respiratory signal and R-peaks in ECG signal respectively. Not all of the 10-minute rest interval at the beginning is shown, as this data is not explicitly analysed in this article.

Experiments consisted of spontaneous breathing for 10 minutes followed by three intervals of guided high-rate breathing (Fig. 1, green shaded areas) with 4 minutes of spontaneous breathing between each. The first guided breathing interval was at 90% of the RHR (started at around 600 s), the second interval (start time is around 1000 s) corresponded to a breathing rate equal to the RHR, and the third interval (around 1400 s) required breathing at 120% of the RHR. The animation guiding breathing ran for 100 complete breath cycles. The total number of guided intervals for the 22 volunteers considered in this work is 66.

Simultaneous recordings of ECG and respiratory signals were performed. According to the data processing procedure described in Methods section, both the breathing and the heart rates were derived in Hertz; however, for illustrative purposes in this paper, the rates are presented in beats-per-minute (BPM). The heart and breathing rates plotted together in Fig. 1 provide a clear picture of the experimental design, with intervals of increased breathing rates, which rise sharply to a rate close to the RHR. The heart rate demonstrates a response to a step change in breathing rates; these step responses will be discussed below.

### Breathing rate as the driving force

**Figure 2:**
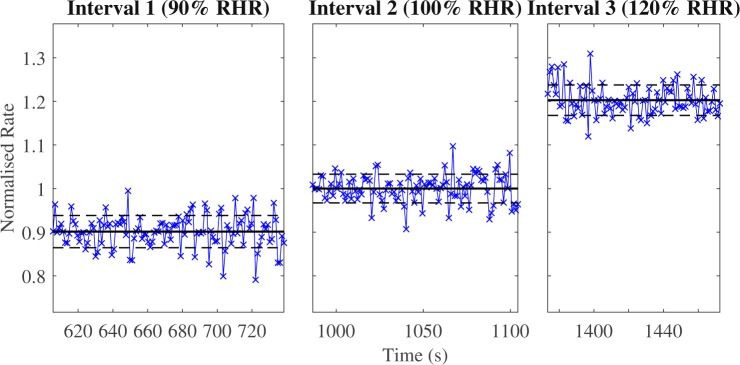
Instantaneous breathing rates, normalised by the mean breathing rate of interval 2, are shown by marker ‘x’. The dashed black lines represent the standard deviation of the rate, while the solid black line is the mean breathing rate for that interval. Assuming a volunteer follows the metronome well, the range between standard deviation lines will be small. The normalisation demonstrates the proportional rate of breathing relative to RHR. Data from volunteer 11.

Owing to the design of the experiment, the breathing rate during guided intervals was intended to be constant. However, experiments demonstrated that individuals were unable to follow the metronome’s rate exactly, so there was a variability in instantaneous breathing rate. Additionally, swallowing or coughing were observed in a few cases. However, the mean breathing rates matched the guided values set by the metronome. Figure 2 highlights how closely volunteers followed the metronome: relative to the normalised interval 2 (100% RHR), the mean rates for interval 1 and 3 are very close to values of 0.9 (90% RHR) and 1.2 (120% RHR), as intended by the experimental procedure. For this volunteer (Fig. 2), the deviation from the mean for each interval is less than 4%. The mean and standard deviation of the breathing rate for all intervals and volunteers are shown in Table SI1 of Supporting Information (SI). For most intervals, the standard deviation is less than 10%. The standard deviation of the breathing rate defines the minimal possible step increments between guided breathing rates. The values for the standard deviation of breathing rates obtained for our cohort confirm that the selected 10% and 20% incremental changes with respect to the RHR guarantee a statistically significant change in the mean value of the breathing rate between intervals of guided breathing.

The Shapiro-Wilk normality test showed that for 33 out of 66 guided intervals, the breathing rate is normally distributed. The deviations from normality are mainly associated with coughing and/or swallowing. The Kwiatkowski–Phillips–Schmidt–Shin (KPSS) test demonstrated that nearly all (63 out of 66) intervals are trend-stationary. Thus, the stochastic component in guided breathing rate can be represented as a Gaussian random process, and the breathing signal itself corresponds to stochastic quasi-harmonic oscillations with a constant amplitude and a variable frequency (see Fig. SI1 in SI).

### Heart rate response to the step change in breathing rate

The mean and standard deviation of the heart rate for all intervals and volunteers are shown in Table SI2 of SI. The variability of this data is significantly stronger than that of the breathing rate data. This can be explained by the nonstationary dynamics of heart rate. Conversely to the guided breathing rate, the KPSS test demonstrated that for 63 out of 66 high-rate breathing intervals, instantaneous heart rate is non-stationary. Furthermore, the Shapiro-Wilk test showed that 49 out of 66 heart rate intervals are not normally distributed. Note that the intervals with a 120% breathing rate, which were the intervals expected to display synchronization, did not correlate directly to the intervals whose heart rate was normally distributed.

The noticed non-stationarity is linked to transient adaptation periods which were observed for most guided intervals, with the heart rates rising to levels disproportionate to the prescribed breathing rate, forming a ramp response. Adaptation was particularly visible during the first interval of high-rate breathing (Fig. 1). Regardless, assuming a volunteer relaxed and continued following the breathing metronome, their heart rate adjusted accordingly. This transient period is less pronounced in the subsequent second and third intervals.

**Figure 3:**
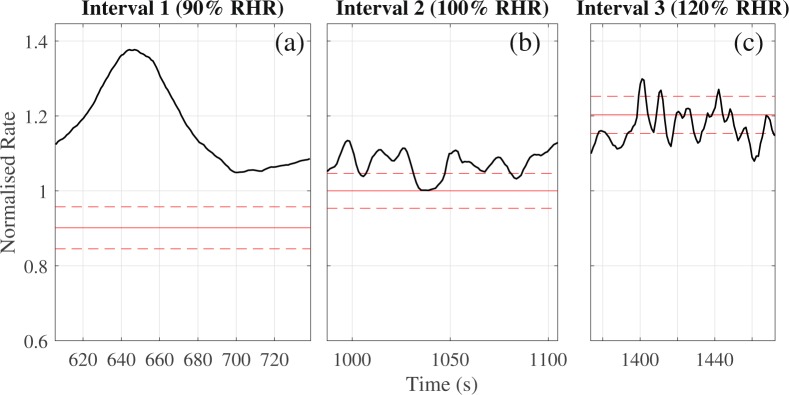
Trends in heart rate during the intervals of guided breathing. Black curves correspond to the trends. Red lines specify the mean value (solid line) and standard deviation (dashed lines) of the breathing rate for each interval. All data normalised by the mean breathing rate of interval 2. The intended heart rate response should mean the black curve falls within the red dashed lines for as much of the interval as possible. Data from volunteer 11.

To analyse the transient response, a slow trend of the heart rate was calculated via a moving average technique described in the Methods section. A variety of trend patterns was observed (Fig. SI2 in SI) and for some intervals there was no trend. In the example presented in Fig. 3, the first interval demonstrates an overshooting response with an initial heart rate increase followed by a decay; this behaviour was typical for our cohort (examples of these plots can be found for all volunteers in Fig. SI2 in SI). The patterns for the second and third intervals were more complex, but the majority included a transient increase of the rate. Rough estimations showed that the duration of this transient increase in the heart rate lasted between 10 and 100 seconds. This observation questions some results^19–21^ where the whole interval of guided breathing was around 30 seconds. For some intervals, the heart rate seemed to begin to tend to a steady state value after the initial adaptation. However, there was no clear steady state observed and for the majority of cases, the heart rate continued to diffuse. In fact, such wandering dynamics are a feature of heart rate^22^ and ought to be considered when analysing synchronization.

### Synchronization

**Figure 4:**
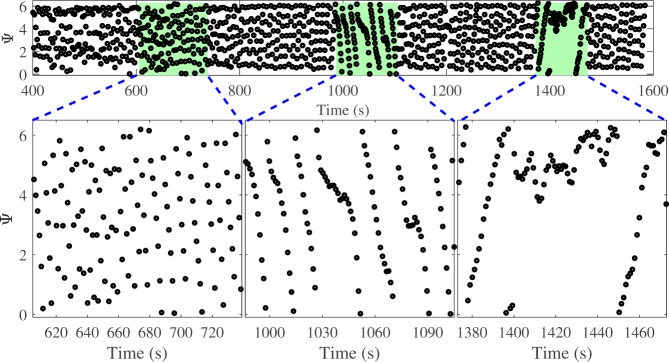
Synchrogram for volunteer 11. Shaded regions correspond to the regions of guided breathing. Phase Ψ is shown in radians.

An example of a synchrogram^6^ encompassing all guided respiration intervals and spontaneous rest periods is shown in Fig. 4, where Ψ represents the relative phase (see Methods section) of the respiratory signal. An episode of phase synchronization with the ratio 1:1 is visible as a plateaued line between 1400 s and 1450 s during the third interval of guided breathing, where the rate is set to 120% of the RHR. During this episode, wandering of the heart rate is limited and the heart rate fluctuates around a particular value (Fig. 3(c)). Before and after this episode the heart rate shows a diffusive behaviour.

For 18 of the 22 volunteers, CRS occurred within the third interval, when the guided breathing rate was higher than RHR. For four volunteers (number 2, 10, 20 and 21), episodes of synchronization were observed for the second interval when the breathing rate was intended to be equal to the RHR. An additional analysis of heart rate during the 10-minute rest interval prior to guided breathing suggests that the RHR value calculated for these was potentially too high, thus for this second interval breathing rate was above the actual RHR. Thus, for all volunteers CRS was observed when breathing rate was higher than RHR. In many cases, more than one episode of CRS was observed within the same time interval. These episodes were automatically identified by the synchronization index^6^ *λ* and the bounded phase difference *φ* as described in the Methods section. The longest episode was singled out and the total duration of all episodes in the given interval was calculated. All results are summarised in Table 1 with times given to the nearest second. The CRS durations calculated by the two methods produced close values. For most volunteers, the longest episode was extended, with durations varying from 20 to 80 seconds, corresponding to 30% to 98% of the whole interval of guided breathing.

**Table 1:** Duration of the longest synchronization episode in seconds (the percentage duration with respect to the whole interval of controlled breathing is shown in brackets) summarised for both methods of episode identification: bounded phase difference (φ ≤ 2π) and synchronization index (λ > 0.7).

Volunteer	1-M	2-F	3-F	4-F	5-M	6*-M	7*-M	8*-M	9*-M	10*-M	11*-M
*φ* ≤ 2*π*	20 (29%) 53 (78%)	81 (98%)	6 (9%) 18 (28%)	17 (22%) 25 (33%)	52 (60%)	52 (74%)	64 (89%)	58 (59%)	66 (65%)	41 (46%)	41 (40%) 72 (71%)
*λ* > 0.7	24 (35%) 35 (51%)	83 (100%)	6 (9%) 10 (16%)	11 (14%)	46 (53%)	15 (21%) 44 (63%)	59 (82%)	49 (50%)	23 (63%)	46 (52%) 84 (94%)	55 (54%) 78 (76%)
	12*-M	**13-F**	**14-M**	**15-M**	16*-F	17*-M	**18-M**	**19-F**	**20-F**	**21-F**	22*-M
*φ* ≤ 2*π*	49 (55%)	29 (35%) 44 (54%)	37 (43%)	25 (25%)	41 (43%) 75 (79%)	40 (35%) 74 (80%)	32 (40%) 49 (60%)	42 (50%) 72 (86%)	31 (33%) 46 (49%)	39 (41%) 58 (60%)	29 (33%) 50 (57%)
*λ* > 0.7	50 (56%) 58 (65%)	26 (32%) 66 (80%)	36 (21%) 51 (56%)	18 (18%) 49 (49%)	37 (39%) 72 (76%)	35 (38%) 58 (62%)	27 (33%) 45 (56%)	41 (49%) 61 (73%)	26 (28%) 44 (47%)	59 (61%) 76 (79%)	25 (28%) 53 (60%)

Where more than one episode occurred, the total time (and corresponding percentage) was calculated and is listed below the time of the longest episode. An asterisk * denotes high fitness volunteers. M or F are used in conjunction with volunteer number to idenitfy male or female.

**Figure 5:**
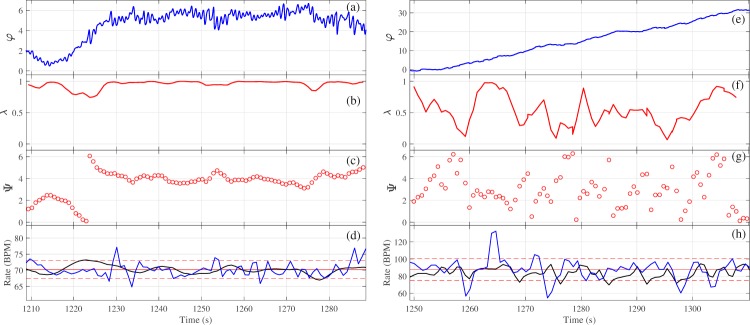
Synchronization measures for volunteer 2 (left) and volunteer 3 (right). Figures **(a**,**e**) show the phase difference, figures (**b**,**f**) show the synchronization index, figures (**c**,**g**) show the synchrogram, and figures (**d**,**h**) show smoothed heart (black line) and respiratory (blue line) rates. In figures (**d**,**h**) red lines specify the mean value (solid line) and standard deviation (dashed lines) of the breathing rate for each interval.

One volunteer (number 3) had very short CRS episodes. The dynamics of the phase difference and rates for the third interval for this volunteer and volunteer 2 are shown in Fig. 5 (similar comparison plots can be found for all volunteers in Fig. SI3 in SI). Interpretation of these plots allows for visualisation of the durations specified in Table 1. The top panel (plot (a) and (e) in Fig. 5) demonstrates the phase difference between heart rate and breathing rate. An oscillation of the phase difference in a limited range less than 2 π, or the phase difference close to a constant value for an extended period is indicative of phase synchronization between the two signals. The duration of synchronization episodes for different volunteers are shown in Table 1. The second panel (plot (b) and (f)) shows time dependence of the synchronization index. A value of the index close to one represents 1:1 synchronization between two oscillating signals. Extended episodes above the experimentally-justified threshold of 0.7 determines the value of *λ* in Table 1. The third panel (plots (c) and (g)) shows the synchrogram for the entire interval of high-rate breathing. During the phase synchronization points on synchrogram demonstrate a plateau. Such plateaus represent one signal’s phase not changing by more than an entire period relative to the phase of the second signal. The final panel (plots (d) and (h)) are a representation of the heart and respiratory rates for a comparison of instantaneous rates during episodes of synchronization with dynamics of phases. The dashed red lines represent the high variability of breathing rate even for controlled breathing- the larger this range, the more variable the breathing rate and thus the worse a volunteer maintained a constant rate. The solid red line is the average breathing rate, and the blue line demonstrates the dynamics of the instantaneous breathing rate throughout the interval. The black line in plots (d) and (h) corresponds to heart rate with removed high-frequency oscillations via applying moving average techniques. During episodes of phase synchronization, the black line is expected to fall wholly between the dashed red lines, representing the fact that the variability of heart rate is contained within the variability of breathing rate.

In Fig. 5, for both cases the heart rates (Fig. 5(d,h)) are visually close to the breathing rates for the whole interval, but the phase dynamics (Fig. 5(a,e)) are remarkably different. The phase difference *φ* (Fig. 5(a)) is limited and nearly constant for volunteer 2, whereas it increases monotonically for volunteer 3 (Fig. 5(e)). This significant difference between phase and rate dynamics stresses the importance of the use of qualitative approaches such as phase description for the analysis of synchronization for signals with stochastic and/or nonstationary components.

**Figure 6:**
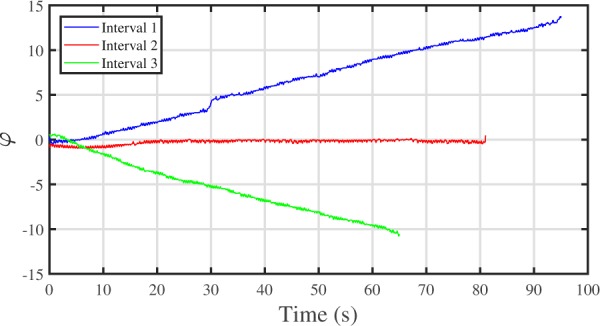
Phase differences *φ* for three guided breathing intervals are shown for volunteer 2. Blue, red and green curves correspond to the first (90% RHR), second (100% RHR) and third (120% RHR) intervals respectively. Phase differences are normalized by 2*π*.

As mentioned, CRS episodes were observed in the second interval (rate intended to be equivalent to RHR) for four volunteers. Therefore, their third interval corresponded to a breathing rate significantly higher than the RHR. Time evolution of the phase difference *φ* for all three intervals is shown in Fig. 6 for one of these volunteers. The phase difference *φ* increases monotonically with time during the first interval, since the heart oscillations are faster than breathing. Limited and nearly constant phase difference during the second interval demonstrates the manifestation of synchronization effect. For the third interval the situation is the opposite and the phase difference decreases monotonically. Note that for all other volunteers, the third interval corresponded to the synchronization interval, and thus a limited phase difference.

There is a clear difference in duration of synchronization episodes between the results for athletes (highlighted by an asterisk) and non-athletes (Table 1). It should be noted that most of the athletes had significantly lower RHR, around 50 BPM (Table SI1 in SI), when compared to other volunteers, and thus would be breathing at lower rates during intervals of guided breathing. This leads to a biasing problem within the design of the experiment - the fitter an individual, and the lower their RHR, the slower they needed to breathe, despite likely being more capable of maintaining higher breathing rates than non-athletes. All athletes had long synchronization episodes. Non-athletes’ results are less consistent; however, the longest episode of synchronization in the cohort was observed for a non-athlete (volunteer 2).

As 9 of the 10 athletes within the study are male, an overall comparison of synchronization episodes between male and female participants is not necessarily representative of an evenly distributed population, and would be inherently biased by the difference between athletes and non-athletes discussed above. When considering non-athletes however, there are 5 males and 7 females. From Table 1 it can be seen that no difference exists between male and female results for the 12 non-athletes. Episodes of synchronization and total durations are of comparable length. Coincidentally, both the longest and shortest episodes of synchronization were female (volunteer 2 and 3, respectively), with clear differences between these results demonstrated in Fig. 5.

Although measures of synchronization considered in this research have successfully identified episodes of CRS, it is important to stress that these episodes could still be coincidental, without any cardio-respiratory interaction. Indeed, it has been discussed above that the heart rate demonstrates wandering (diffusive) dynamics and changes in a wide range. Due to this diffusivity, when breathing and heart rates are close to each other for a period of time, their average rates are nearly equal, and no differences between the rates would be clearly seen during these short periods. This, in turn, would mean that all measures, i.e. synchrogram, synchronization index, and phase difference, would identify these time periods as episodes of synchronization even in the absence of a true cardio-respiratory interaction. Therefore, in this work we additionally demonstrate that the episodes that we observed were not coincidental by employing surrogate data.

**Figure 7:**
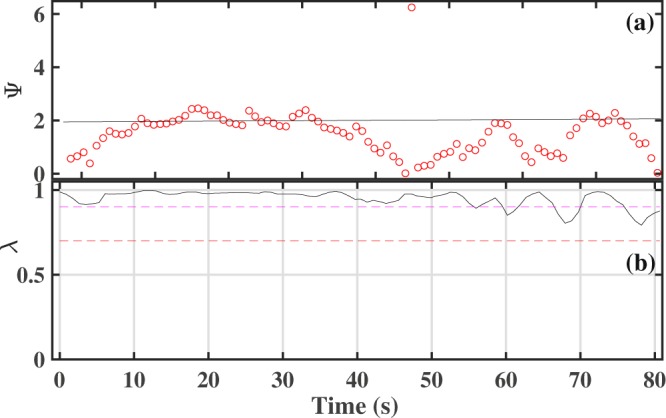
Synchrogram (**a**) and synchronization index (**b**) for surrogate data; the mean is 70 BPM and the standard deviation is 3%. Red and magenta dashed lines correspond to *λ* = 0.7 and *λ* = 0.9 respectively.

**Figure 8:**
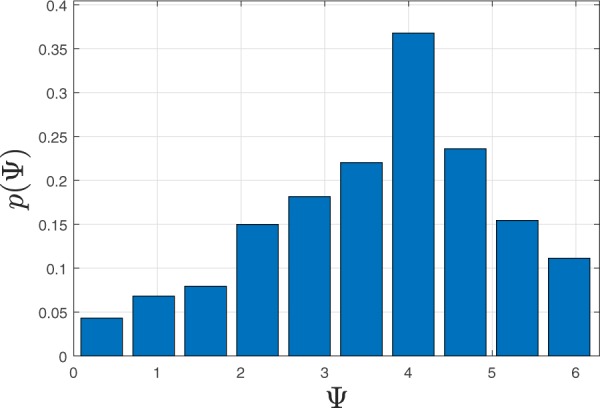
Probability density *p*(Ψ) for experimental data is shown in a bar chart. Phase Ψ is shown in radians.

Let us consider surrogate breathing and heart rates generated using random, normally distributed data, and derive synchrogram and synchronization index for this data. The mean values (70 BPM) and standard deviations (3%) of breathing and heart rates are selected to be equal and correspond to two different random time series. These rates have been converted to instantaneous periods, as described in the Methods section, which leads to two surrogate time series: one of R-peaks of an ECG signal and the other of maxima of a breathing signal. Then the same signal processing techniques were applied as to the experimental data, and the phase difference Ψ was calculated together with the synchronization index *λ* (Fig. 7). It can be seen that the phase Ψ (Fig. 7a) is nearly constant (Ψ_*C*_ ≈ 2) for a long time interval and the synchronization index *λ* (Fig. 7b) is larger than the threshold value (0.7) for all of the interval. Therefore, synchronization episodes for these surrogate data are clearly observed. It is important to note that for the surrogate data, the nearly constant value Ψ_*C*_ of phase Ψ on the synchrogram is a random value, despite all measures showing episodes of synchronization. For example, in Fig. 7 the phase is around 2 (Ψ_*C*_ ≈ 2), but would take a different value for another set of surrogate data. Consequently, for synchronization observed by chance, the distribution of *p*(Ψ) for a set of measurements must be uniform since the time series of rates are fully independent. On the other hand, a difference from a uniform distribution *p*(Ψ) would indicate the presence of coupling between the cardio and respiratory systems. In Fig. 8, the distribution *p*(Ψ) derived from our experimental data for all 22 intervals of guided breathing with synchronization episodes from all volunteers is shown. The values of Ψ were selected from synchrogram plots when *λ* > 0.9. The threshold value was increased versus experimental analysis to highlight episodes of strongest interaction (note that other threshold values, e.g. 0.7, lead to a similar shape of the distribution). The distribution (Fig. 8) is skewed and it has the most probable value of 4; Ψ_*C*_ ≈ 4. This result indicates that the phase locking in our experimental data is observed for a particular value of Ψ and therefore synchronization episodes are not coincidental and result from a true cardio-respiratory interaction.

## Discussion

Respiratory and heart dynamics during guided high-rate breathing, measured and analysed for 22 volunteers, showed that the high-rate breathing is sufficiently adaptable and can be accurately controlled. This was demonstrated by the stationarity and normality of the breathing rate time series during the intervals of guided breathing. The observed variability of the breathing rate defines the limitations on the size of the incremental steps for high-rate guided breathing recordings. Conversely, heart rate responses showed high variability, and were non-stationary and not normally-distributed. A long transient period of adaptation to a step-change in breathing rate was observed for the majority of intervals. The transient periods were shorter for the intervals with episodes of synchronization. Additionally, the heart rate demonstrated diffusive dynamics after the adaptation period.

Both methods of synchronization analysis, i.e. synchronization index and bounded phase difference, have confirmed the presence of at least one episode of phase synchronization with a ratio 1:1 in all 22 volunteers, and showed high correlation between the methods. 8 of 22 volunteers had a single episode of 40 seconds or more which was identified by both methods. 20 of the 22 volunteers demonstrated CRS identified by at least one method for a total time of 40 seconds or more. All athletes had an extended total duration, in excess of 40 seconds. Similar durations of synchronization episodes were reported for spontaneous breathing rates, however these episodes were extracted from significantly longer (around 8-hour) recordings^10,12^.

Differences between athletes (volunteers who perform high-intensity exercise requiring rhythmic control of breathing) and non-athletes is an aspect of particular interest and potential applicability. If individuals of higher fitness and autonomic health demonstrate longer CRS episodes, there is the possibility to utilise this non-invasive measure for tracking a person’s athletic development.

A preferable range of values of phase Ψ_*C*_ in the synchrogram during synchronization suggests entrainment between the two physiological signals via heart rate locking by a constant elevated breathing rate. Such an interaction occurring at breathing rates exceeding the heart rate at rest, i.e. at normal breathing rate, is in agreement with suggestions from previous research^20^. However, relatively long periods of adaptation, the stochasticity of the breathing rate signal even when guided, the diffusive dynamics of heart rate, and intra-volunteer variability in the duration of synchronization episodes observed in this research show a much more complicated picture of cardio-respiratory interaction than previously reported^19–21^. The developed methodology is based on using multiple statistical and graphical measures to identify intervals of synchronization, and mitigates the possibility of highlighted interactions being coincidental, leading to a robust analysis procedure. Investigating cardio-respiratory interaction through rigorous analysis utilising the phase approach is perhaps the key factor in this complex but comprehensive picture. Another factor is a different experimental setup, considering CRS in a lying rather than standing body position, which reduces the influence of the visual neural centre and mechanical cardio-respiratory coupling. Further investigation is required to compare the durations of CRS in various body positions and the influence of a strong visual signal such as a flashing light on CRS.

There is a significant volume of previous research related to CRS for a low breathing rate. In contrast to high-rate breathing where it is assumed^20^ that CRS results from the links in the neural centres, low-rate CRS should result from peripheral coupling between cardio and respiratory neural centres^16^. The mechanism of synchronization is therefore assumed to be different depending on the breathing rate. However, our results demonstrate that CRS at both high and low breathing rates^7,10^ is linked to a common regulatory mechanism since the synchronization is more pronounced in physically fitter people for both rates. Thus, the question about the origin of CRS for high breathing rate stands: whether CRS is the result of coupling between the two neuronal centres in the medulla oblongata (as publication^18^ suggests) or of the mechanical force of breathing at a high rate. Let us stress that CRS at a 1:1 ratio, i.e. at a high breathing rate, is a robust phenomenon which can be analysed for each volunteer, whereas synchronization for a low breathing is an elusive effect having a transient appearance^10,12^. For understanding common features and differences in cardio-respiratory coupling during low-rate breathing and high-rate breathing, further investigation is needed by applying a range of breathing regimes to the same individual.

## Methods

### Experimental setup and measurements

22 volunteers between 18 and 30 years of age took part in the measurements. 10 people were characterized by high fitness levels, all being rowers (6), cyclists (2), swimmers (2), or weightlifters (8), with some crossover. Such physical activities require rhythmic control of breathing throughout, potentially enhancing the connections within the cardio-respiratory system and peripheral control centres. Let us note that CRS was first reported in work^7^ for a small group of elite swimmers. The other 12 volunteers have a range of physical fitness, with some doing daily exercise, some infrequent exercise, and others doing none. All had at least average fitness levels. Data for the detection of CRS was collected using a BIOPAC physiological signal monitoring system^23^. The device enabled simultaneous, non-invasive recording of the ECG and respiratory signal. The ECG was measured by electrode stickers in lead II configuration^2^. The respiratory signal was recorded with a respiration belt transducer. The ECG and respiratory signal were recorded with sampling rate of 1000 Hz. To perform measurements, ethical approval was granted by the Biomedical and Scientific Research Ethics Committee (BSREC) at the University of Warwick (REGO-2013-565). This includes compliance with the Ethical Principles for Medical Research on Human Subjects set by the Declaration of Helsinki by the World Medical Association. All volunteers provided informed consent after reading the information leaflet and receiving a briefing from the lead investigator.

As suggested in work^20^, for CRS with ratio 1:1 to occur the breathing rate must be equal to or exceed the RHR. The breathing rate was chosen to be a control signal as the heart is an autonomous system and cannot be consciously controlled independently of other processes. Upon visiting the laboratory, volunteers had sufficient rest time in a lying position (20–30 minutes before recording started) to ensure their RHR and breathing rates were as natural and relaxed as possible. The individual values of RHR were derived from the last 4 minutes of the rest time. The experiment was conducted with volunteers lying down with an elevated thorax and head. This position allowed the volunteers to view the sinusoidal display metronome, which was designed to guide the rate of breathing. The animation determined when to breathe in and out, with the approximate duration of each breath fixed.

### Rate and phase of signals

The raw data was post-processed to extract each peak, representative of individual breaths and heart beats, using an event-based approach^6^. The R-peak in the ECG signal was used to identify the time moment *t*_*i*_ of a heart beat via custom software with an identification error of 0.001 seconds. The difference between two subsequent beats gives an instantaneous period, *T*_*i*_ = *t*_*i*_ − *t*_*i*−*1*_, and its reciprocal defines the instantaneous rate (frequency) *r*_*i*_ and the corresponding time series of the heart rate *r*_*i*_ (*t*_*i*_). The phase of the heart signal *φ*_*h*_ (*t*_*i*_) between two consecutive events changes by 2*π*, i.e. *φ*_*h*_ (*t*_*i*_) = 2*πi*. The phase, *φ*_*h*_ (*t*), at an arbitrary time moment was identified via linear interpolation.

For the identification of a single breath, the maximal value of each breath cycle was used. Then, following the approach described for ECG signal, the instantaneous breathing rate was calculated together with the corresponding phase time series. Due to physiological artefacts in the recordings of respiratory signals at higher breathing rates, the identification of the maximal values (peaks) can lead to errors. Therefore, a different approach for calculating breathing phase *φ*_*b*_ (*t*) was used for the guided intervals of high-rate breathing. The respiratory signal was filtered via a second-order pass band Butterworth filter with cut off frequencies of 0.1 Hz and 3 Hz. An example of the initial and filtered respiratory signals are shown in Fig. SI1 in SI. This removed the low-frequency trend and high-frequency fluctuations. After filtering, the Hilbert transform was applied to calculate the time-dependent phase *φ*_*b*_ (*t*).

### Analysis of stochasticity

Significant variability of the breathing and heart rates was observed in the experiments (see Fig. 1 for an example). To characterise the variability of signals, the mean and standard deviation of the rates were calculated, and stationarity and normality tests were conducted. Due to the relatively small data size (around 100 breaths or heart beats per interval), the Shapiro-Wilk normality test was implemented to categorise normality, as this test yields a high statistical power with small sample sizes^24^. Stationarity was described in terms of an underlying trend, using the KPSS null hypothesis test^25^. A significance level of 0.05 was used for both tests.

### Transient response

To analyse the transient response of heart rate to the step change in breathing rate, a moving average technique was applied to extract a low-frequency trend in the heart rate time series. Specifically, the “smooth” heart rate was calculated as the mean value within time windows of 10 heart beats, with the centre of each window located at the given time moment.

### Identification of phase synchronization

To illustrate the effect of phase synchronization, a synchrogram^6^ was used. It is constructed by calculating the phase of a slow signal, *ϕ*_*l*_, at the time moment *t*_*k*_ corresponding to a change of phase of the fast signal by 2*π*, for example at the time moment when an event of the fast signal occurs, and by plotting relative phase Ψ(*t*_*k*_) defined by the following expression: Ψ(*t*_*k*_) = *ϕ*_*l*_ mod 2*π*. Respiratory rate was used as a slow signal and heart rate was used as a fast signal. In the case of synchronization in a *n*:1 ratio, *n* parallel lines are visible on a synchrogram; a single line is an indicator of synchronization in a 1:1 ratio.

To quantify the existence of CRS and to calculate the durations of synchronization episodes during the intervals of high-rate breathing, the phase difference and synchronization index were calculated and analysed as functions of time. The phase difference is *φ*(*t*) = *φ*_*h*_ (*t*)–*φ*_*b*_ (*t*), where *φ*_*h*_ (*t*) and *φ*_*b*_ (*t*) are the phases of the ECG and respiratory signals respectively. When synchronization phenomenon is observed, *φ*(*t*) is nearly constant and is limited by 2*π*. To find the duration of synchronization episodes, the time intervals with phase difference *φ*(*t*) less than 2*π* were identified; epochs longer than 5 seconds were considered episodes of synchronization.

The synchronization index *λ*(*t*) characterizes the strength of synchronization in terms of how close the phase difference is to a constant value. It can be calculated using the following equation^6^:

1 $$\lambda (t)=\sqrt{{\langle \cos (\phi (t))\rangle }_{t}^{2}+{\langle \sin (\phi (t))\rangle }_{t}^{2}}$$

The angular brackets denote an average over a time window with its centre at time moment, *t*. The width of the window was chosen to be 7 heart beats. Full phase-locking with a constant phase difference would correspond to the unitary mean value of index *λ*: *λ* = 1, whereas no phase-locking effect would yield a zero value of *λ*. Since both signals, respiratory and heart, are stochastic, the phase difference is not a constant and fluctuates with time. Consequently, to identify synchronization episodes, an appropriate cut-off value of *λ* was derived experimentally. Namely, a comparative analysis of the dynamics of synchronization index and of the corresponding synchrogram plots led to the threshold value *λ*_thr_ = 0.7. Synchronization indices *λ* ≥ *λ*_thr_ indicate an episode of phase synchronization, and the continuous time interval within which *λ* ≥ *λ*_thr_ corresponds to its duration.

## Supplementary information

Supporting information

## Data Availability

Equipment, methods, and software used are available from the corresponding author. The datasets recorded and analysed during the current study are not publicly available due to constraints within the ethical approval concerning volunteer data protection. Anonymised time series are available from the corresponding author on reasonable request.
