# Supplementary material for: Control of heart rate through guided high-rate breathing
Source: Sci Rep. 2019 Feb 7;9:1545. doi: 10.1038/s41598-018-38058-5 (PMC6367452; doi:10.1038/s41598-018-38058-5)
Supplement: Supplementary file 1 — Supporting information [file 41598_2018_38058_MOESM1_ESM.docx]

**Supporting information**

**Control of heart rate through guided high-rate breathing**

Sean Perry, Natasha A Khovanova*, Igor A Khovanov

*N.Khovanova@warwick.ac.uk

School of Engineering, University of Warwick, Coventry CV4 7AL, United Kingdom

This document contains data for all volunteers.

Tables SI1 and SI2 contain information on the variability of measured physiological signals, with the averages and standard deviation of breathing rate and heart rate shown. The tables include information on sex and fitness level of each volunteer.

A measured and detrended respiratory signal is displayed in Figure SI1, to illustrate the quasi-harmonic nature of this signal.

The plots in Figure SI2 show a variety of patterns in the transient response of the heart rate to the step change in respiration. Some common patterns can be observed here, such as “overshoot” of heart rate to lower breathing rate step changes. A saturation value for heart rate can be seen in many cases. These plots further emphasise the complexity of analysing physiological signals, owing to changing dynamics of heart rate and variability of breathing rate (dashed red lines), even with a guiding metronome.

Figure SI3 combines all the output plots for analysing synchronization. With the corresponding panels, comparison can be made between the different techniques used for identification of phase synchronization.

Table SI1. Mean and standard deviation (SD) of instantaneous breathing rates for the 3 intervals of guided breathing. Standard deviations are presented as percentages of the mean value. Units of mean value are beats-per-minute (BPM). Marker * denotes an athlete. Letters M or F highlights male or female volunteers respectively. Interval 2 can be used to estimate the resting heart rate of individuals. The value of standard deviation for instantaneous breathing rate varies significantly, demonstrating an inability of volunteers to follow the metronome exactly. The variability of the breathing rate underlines a stochastic component in the rate.

|  | **Mean Breathing Rate** *± SD%* (BPM) | | |
| --- | --- | --- | --- |
| Volunteer I | Interval 1: 90% RHR | Interval 2: 100% RHR | Interval 3: 120% RHR |
| 1-M | 67.7 *± 4.3%* | 75.1 *± 3.3%* | 90.1 *± 3.2%* |
| 2-F | 63.1 *± 5.1%* | 70.2 *± 4.6%* | 84.0 *± 4.0%* |
| 3-F | 67.9 *± 7.2%* | 75.6 *± 7.3%* | 87.6 *± 18.4%* |
| 4-F | 58.6 *± 4.4%* | 65.4 *± 8.1%* | 75.4 *± 8.0%* |
| 5-M | 51.6 *± 5.2%* | 59.1 *± 5.4%* | 69.8 *± 3.4%* |
| 6*-M | 66.2 *± 11.6%* | 71.9 *± 8.9%* | 84.2 *± 8.2%* |
| 7*-M | 62.6 *± 8.8%* | 69.8 *± 5.2%* | 84.0 *± 3.2%* |
| 8*-M | 45.1 *± 6.7%* | 49.9 *± 4.0%* | 60.0 *± 3.7%* |
| 9*-M | 45.1 *± 5.1%* | 49.1 *± 4.3%* | 60.0 *± 3.8%* |
| 10*-M | 61.3 *± 4.1%* | 67.9 *± 3.1%* | 81.4 *± 3.9%* |
| 11*-M | 44.2 *± 4.1%* | 49.0 *± 3.3%* | 58.9 *± 2.9%* |
| 12*-M | 50.3 *± 4.3%* | 60.0 *± 3.6%* | 67.7 *± 3.2%* |
| 13-F | 54.8 *± 4.2%* | 60.3 *± 4.6%* | 73.1 *± 3.2%* |
| 14-M | 51.3 *± 1.7%* | 57.0 *± 2.7%* | 68.6 *± 1.3%* |
| 15-M | 46.0 *± 2.2%* | 51.0 *± 2.5%* | 61.2 *± 1.7%* |
| 16*-F | 47.8 *± 2.9%* | 53.1 *± 4.3%* | 63.6 *± 2.2%* |
| 17*-M | 53.4 *± 7.1%* | 59.1 *± 6.3%* | 65.0 *± 9.1%* |
| 18-M | 56.0 *± 5.4%* | 62.7 *± 8.8%* | 75.9 *± 4.0%* |
| 19-F | 50.2 *± 10.2%* | 60.0 *± 5.9%* | 72.4 *± 7.1%* |
| 20-F | 58.7 *± 5.2%* | 64.2 *± 8.5%* | 72.8 *± 4.2%* |
| 21-F | 56.8 *± 4.6%* | 62.8 *± 7.1%* | 76.5 *± 3.4%* |
| 22*-M | 51.4 *± 3.4%* | 57.2 *± 3.0%* | 68.7 *± 2.5%* |

Table SI2. Mean and standard deviation (SD) of instantaneous heart rates for the 3 intervals of guided breathing. Standard deviations are presented as percentages of the mean value. Units of mean value are beats-per-minute (BPM). Marker * denotes an athlete. Letters M or F highlights male or female volunteers respectively. The heart rate exhibits a complex response to the step change in respiration. A trend in the response of heart rate is illustrated in Figure SI2.

|  | **Mean Heart Rate** *± SD%* (BPM) | | |
| --- | --- | --- | --- |
| Volunteer | Interval 1: 90% RHR | Interval 2: 100% RHR | Interval 3: 120% RHR |
| 1-M | 84.7 *± 8.7%* | 87.4 *± 3.9%* | 89.4 *± 8.1%* |
| 2-F | 71.0 *± 3.1%* | 70.2 *± 2.3%* | 73.9 *± 3.8%* |
| 3-F | 82.4 *± 6.9%* | 83.0 *± 7.7%* | 83.9 *± 9.3%* |
| 4-F | 81.0 *± 6.5%* | 79.0 *± 6.1%* | 87.0 *± 4.9%* |
| 5-M | 77.9 *± 16.2%* | 63.9 *± 8.0%* | 67.8 *± 10.0%* |
| 6*-M | 81.1 *± 5.2%* | 82.9 *± 8.9%* | 82.5 *± 5.6%* |
| 7*-M | 87.1 *± 5.7%* | 87.0 *± 6.0%* | 83.6 *± 4.5%* |
| 8*-M | 64.8 *± 9.1%* | 58.0 *± 10.9%* | 58.3 *± 12.0%* |
| 9*-M | 59.5 *± 8.7%* | 61.0 *± 7.5%* | 58.0 *± 5.3%* |
| 10*-M | 71.1 *± 7.3%* | 69.8 *± 3.7%* | 74.0 *± 3.6%* |
| 11*-M | 58.2 *± 12.5%* | 52.7 *± 4.0%* | 57.6 *± 5.0%* |
| 12*-M | 66.1 *±* 6.0% | 61.0 *±* 5.8% | 66.8 *±* 4.7% |
| 13-F | 91.2 *±* 6.9% | 81.1 *±* 4.5% | 76.5 *±* 4.2% |
| 14-M | 68.4 *±* 7.3% | 66.4 *±* 9.6% | 67.8 *±* 6.6% |
| 15-M | 65.6 *±* 6.7% | 62.6 *±* 4.9% | 59.8 *±* 6.7% |
| 16*-F | 71.4 *±* 7.2% | 62.3 *±* 6.6% | 65.3 *±* 4.8% |
| 17*-M | 66.9 *±* 4.1% | 68.2 *±* 3.9% | 65.1 *±* 2.6% |
| 18-M | 87.2 *±* 6.3% | 78.1 *±* 6.2% | 77.6 *±* 5.7% |
| 19-F | 81.9 *±* 7.0% | 77.7 *±* 6.8% | 74.4 *±* 5.5% |
| 20-F | 82.7 *±* 6.3% | 68.3 *±* 7.1% | 64.5 *±* 6.2% |
| 21-F | 67.2 *±* 3.9% | 65.8 *±* 5.0% | 68.0 *±* 2.6% |
| 22*-M | 73.5 *±* 3.3% | 73.0 *±* 3.6% | 71.0 *±* 4.7% |


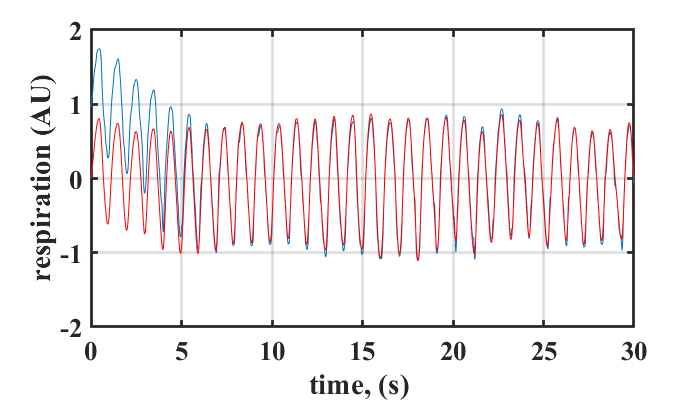


Figure SI1. An example of measured respiratory signal before (blue curve) and after (red curve) trend removing for an interval of guided breathing. The signal can be considered as quasi-monochromatic with frequency fluctuating around a particular value and with nearly constant amplitude.

| 1 | 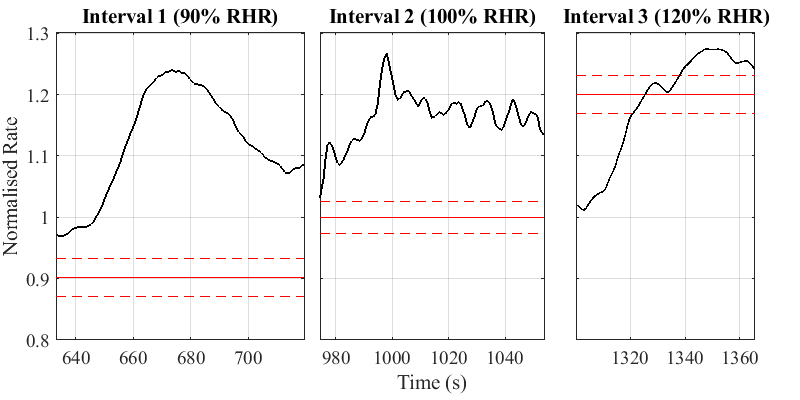 |
| --- | --- |
| 2 | 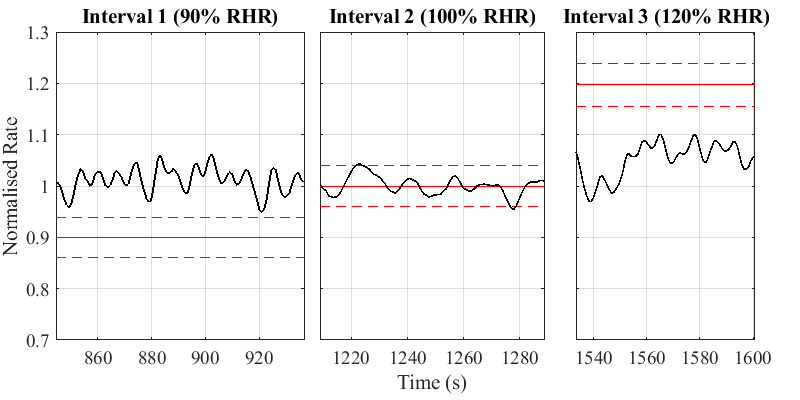 |
| 3 | 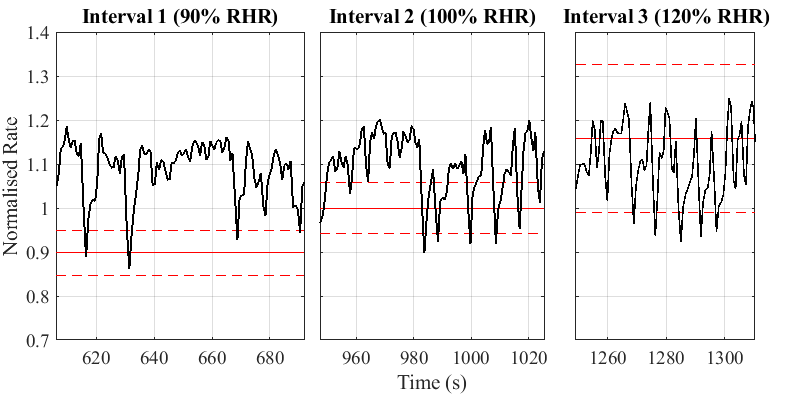 |
| 4 | 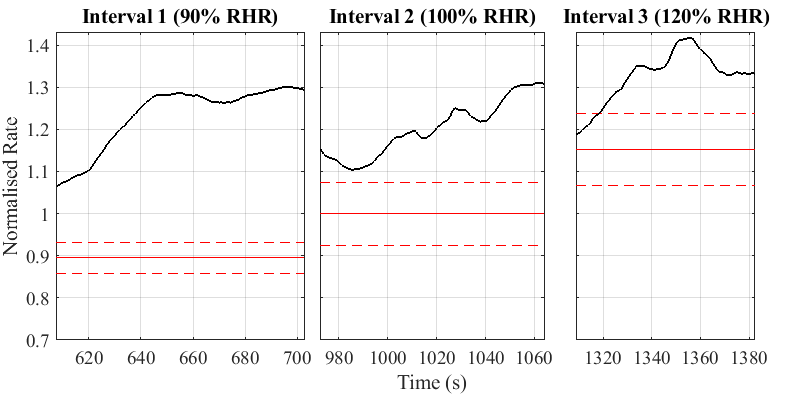 |
| 5 | 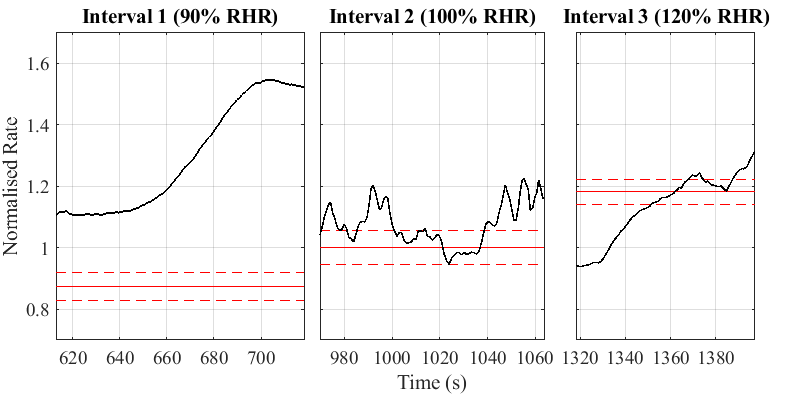 |
| 6 | 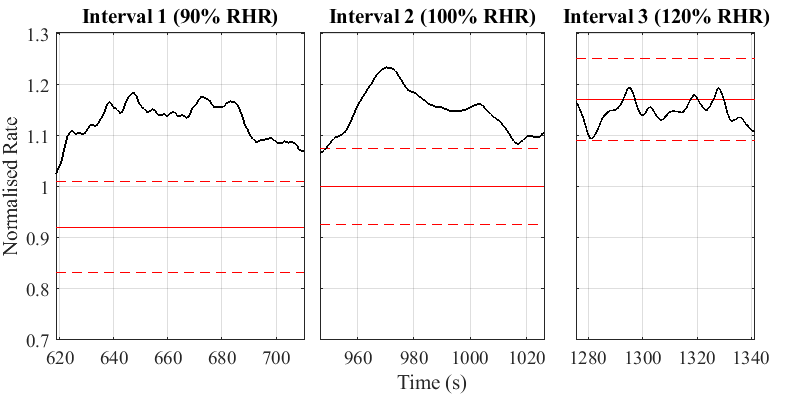 |
| 7 | 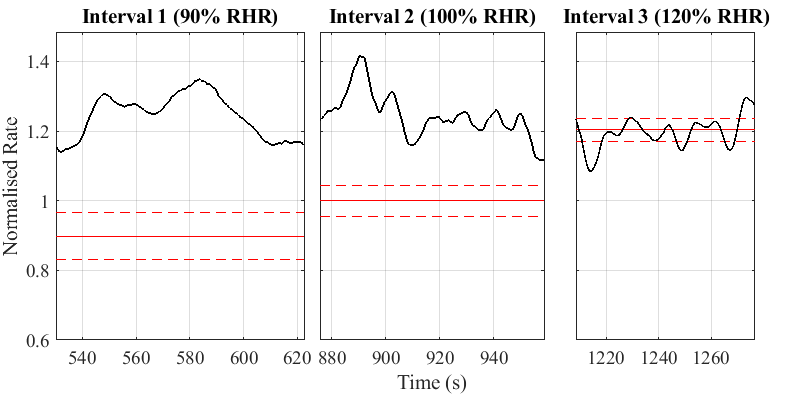 |
| 8 | 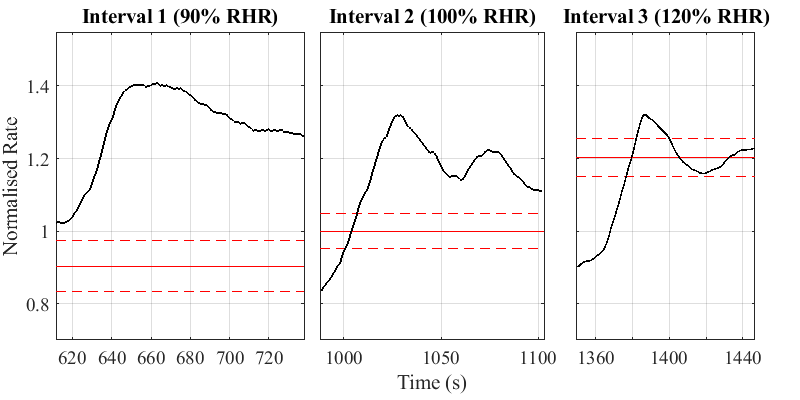 |
| 9 | 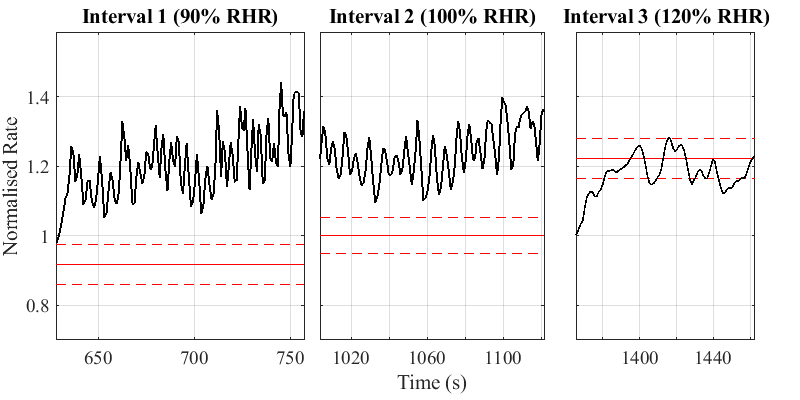 |
| 10 | 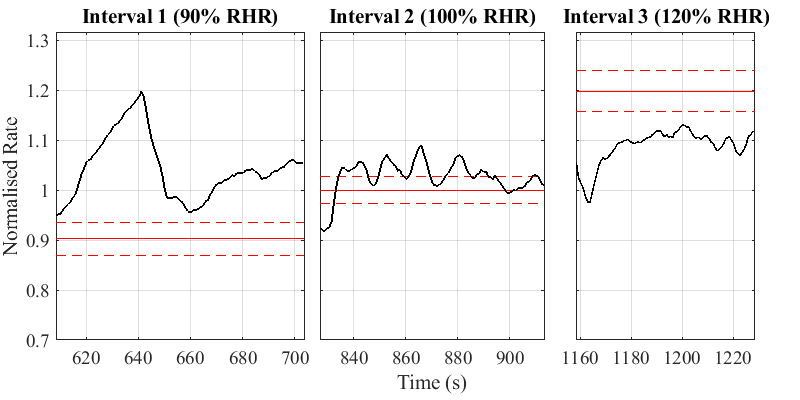 |
| 11 | 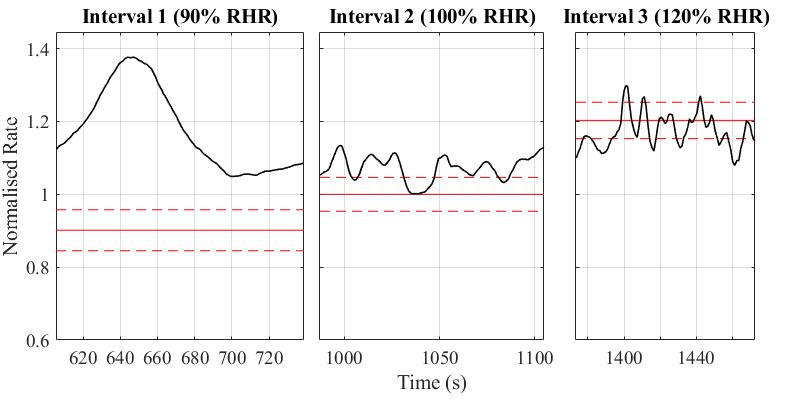 |
| 12 | 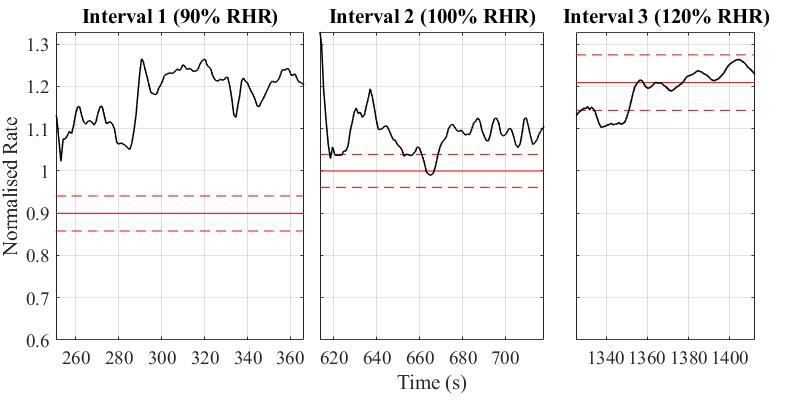 |
| 13 | 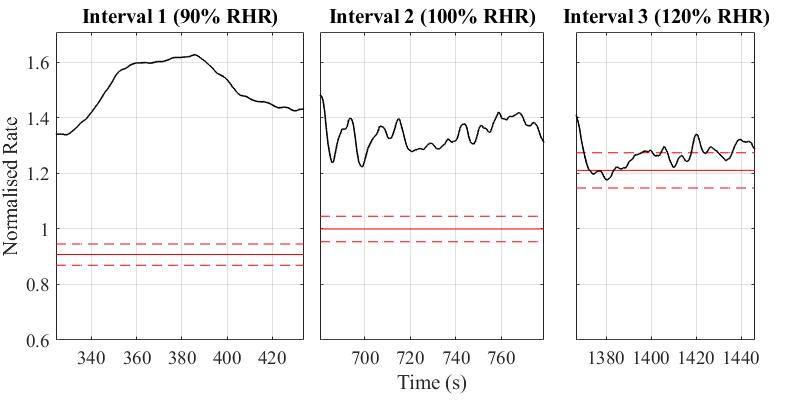 |
| 14 | 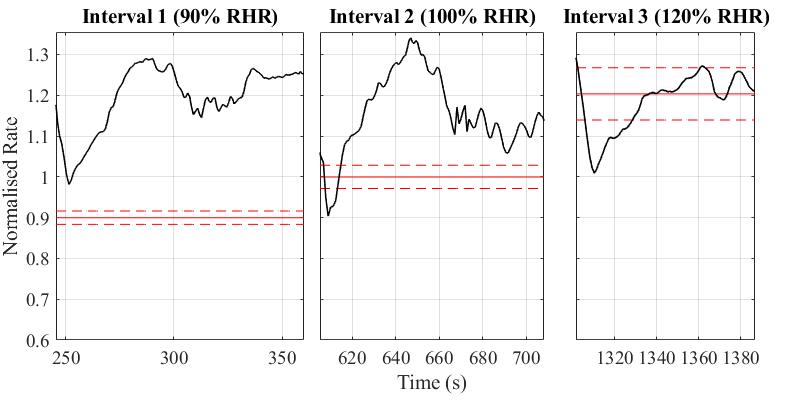 |
| 15 | 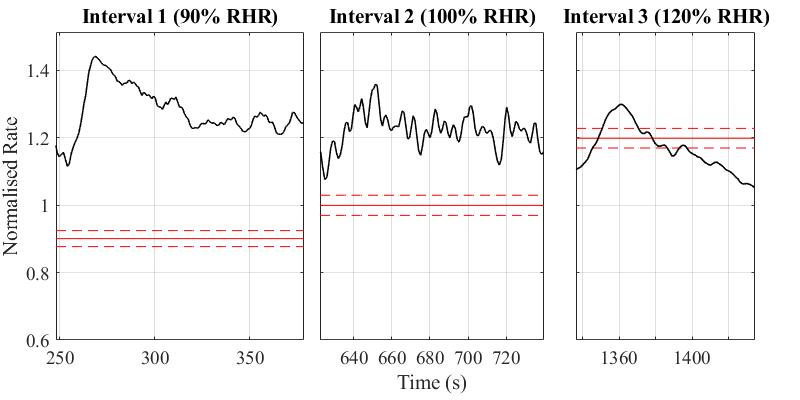 |
| 16 | 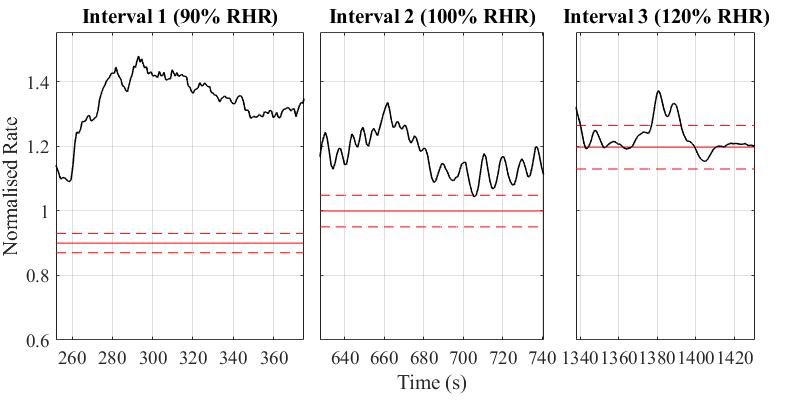 |
| 17 | 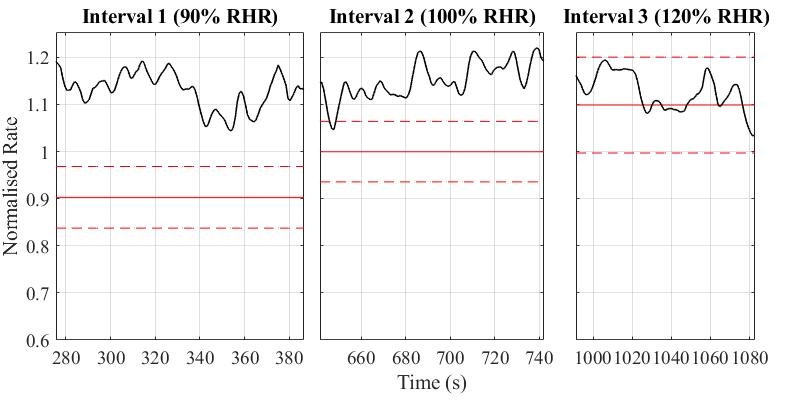 |
| 18 | 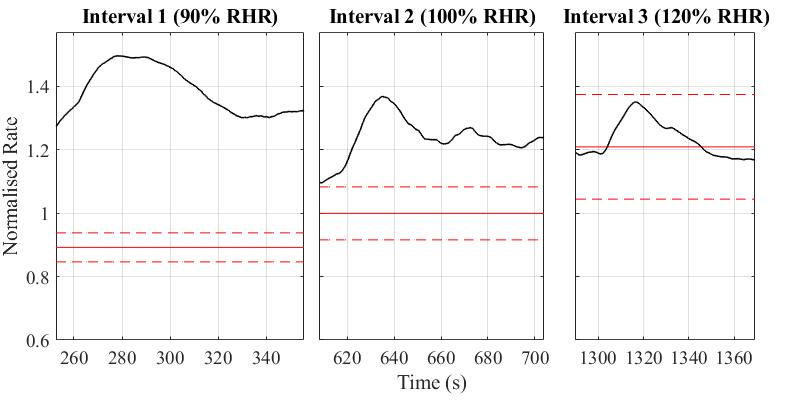 |
| 19 | 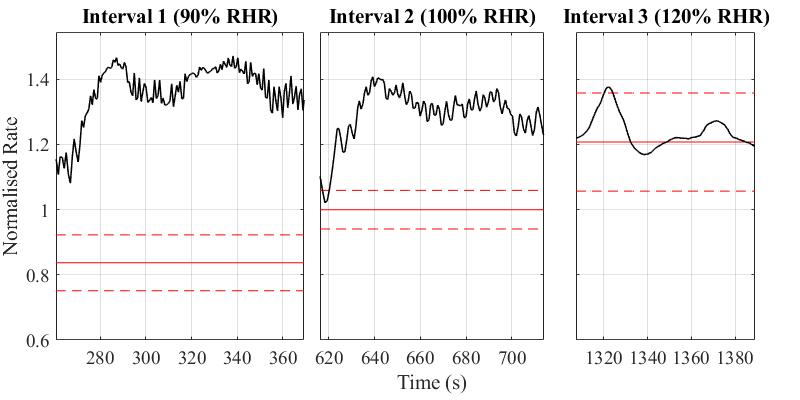 |
| 20 | 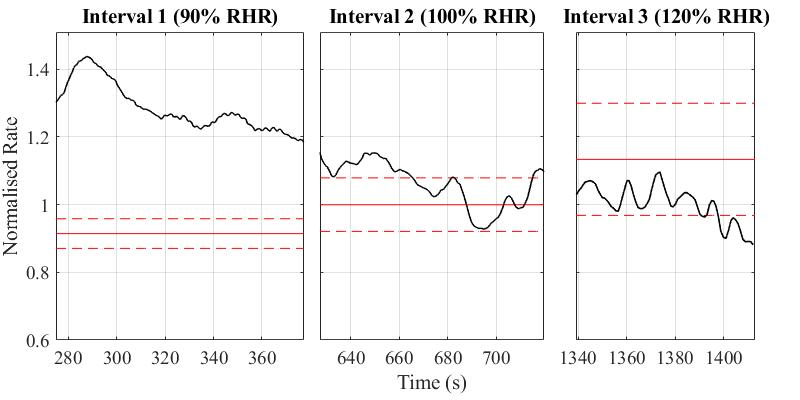 |
| 21 | 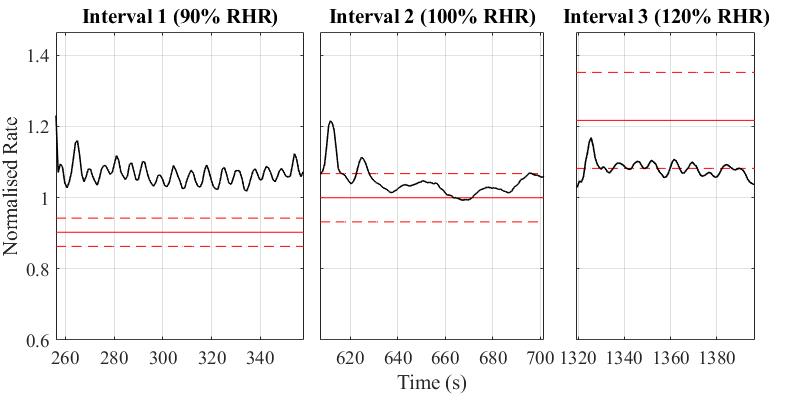 |
| 22 | 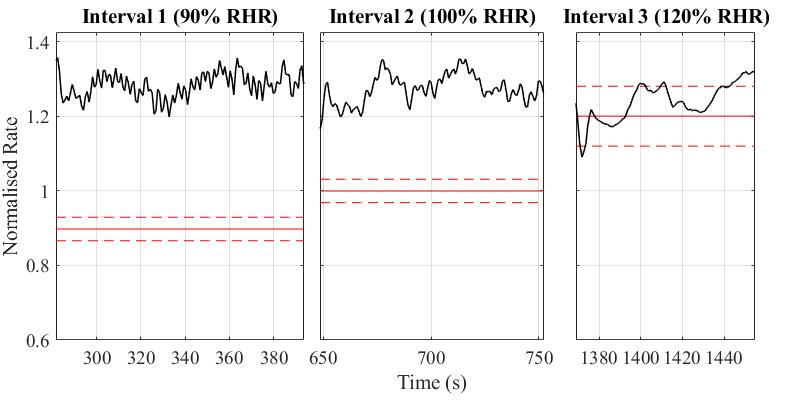 |

Figure SI2. Dynamics of the transient response of heart rate for intervals of guided breathing. Numbers on the left correspond to each volunteer’s numeration. Black curves correspond to the slow trends obtained via a moving average technique applied to instantaneous heart rate. Red lines specify the mean value (solid line) and standard deviation (dashed lines) of breathing rate for each interval. A complex picture is displayed here, as heart rate is often seen to change significantly in response to lower rates of breathing (90% and 100% of RHR). This step response is discussed in the Results section of the main article (subsection: *Heart rate response to step change in breathing rate*). However, no single reaction is common for all volunteers, demonstrating the added complexity of explaining differences in dynamics for even a small cohort of volunteers. Episodes of 1:1 synchronization could be observed during intervals where an extended proportion of the black curve (heart rate) is contained within the variability lines (dashed red) for the breathing rate. Assuming comparable variability, the two signals will be changing synchronously in time.

| 1 | 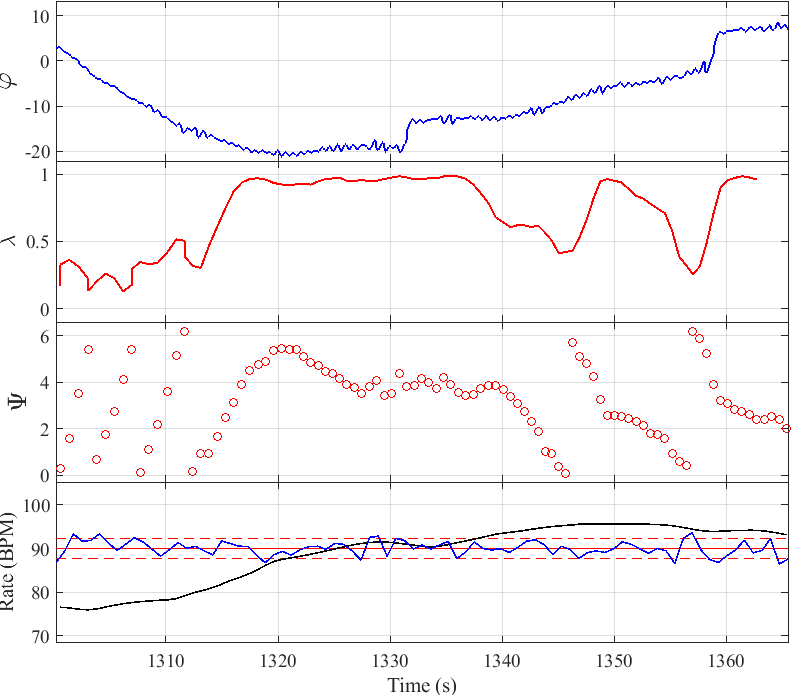 | 2 | 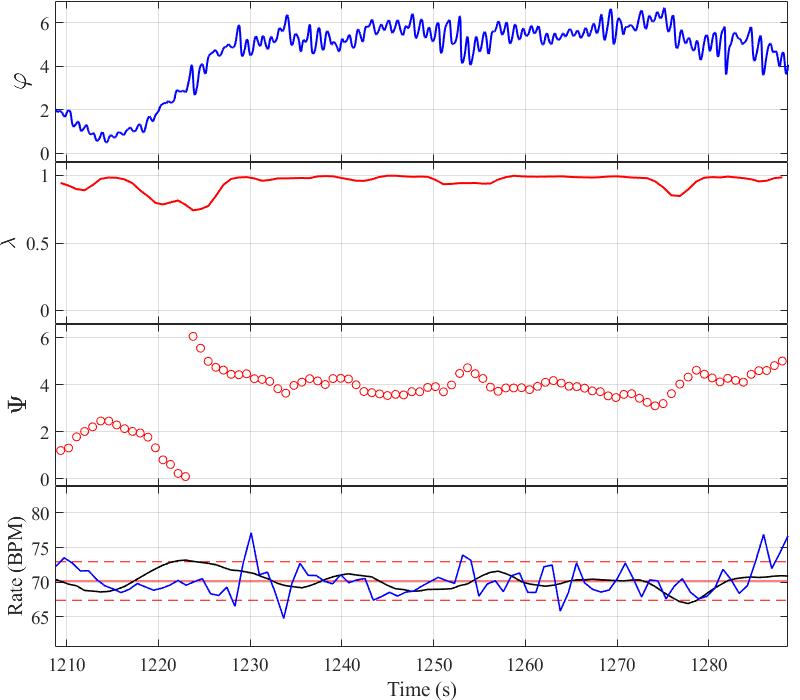 |
| --- | --- | --- | --- |
| 3 | 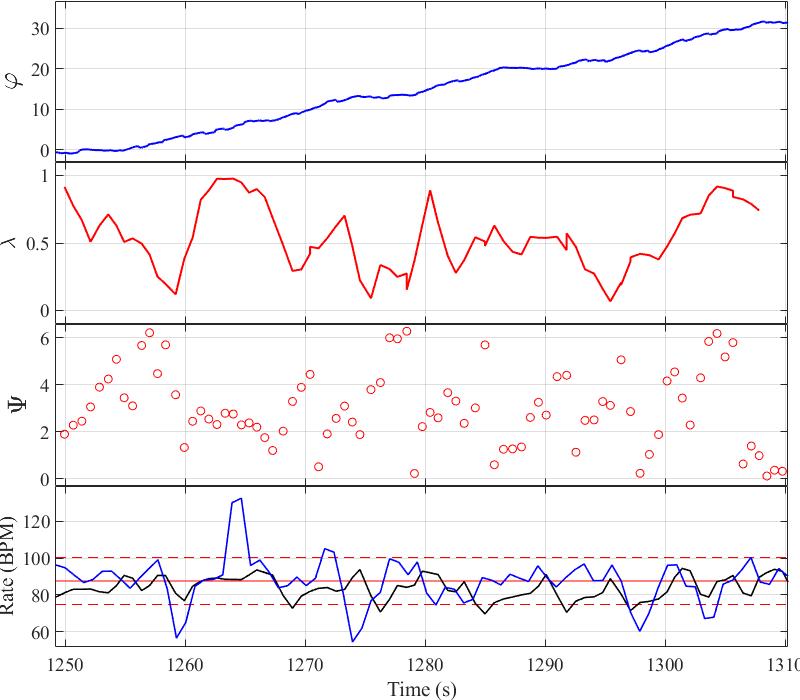 | 4 | 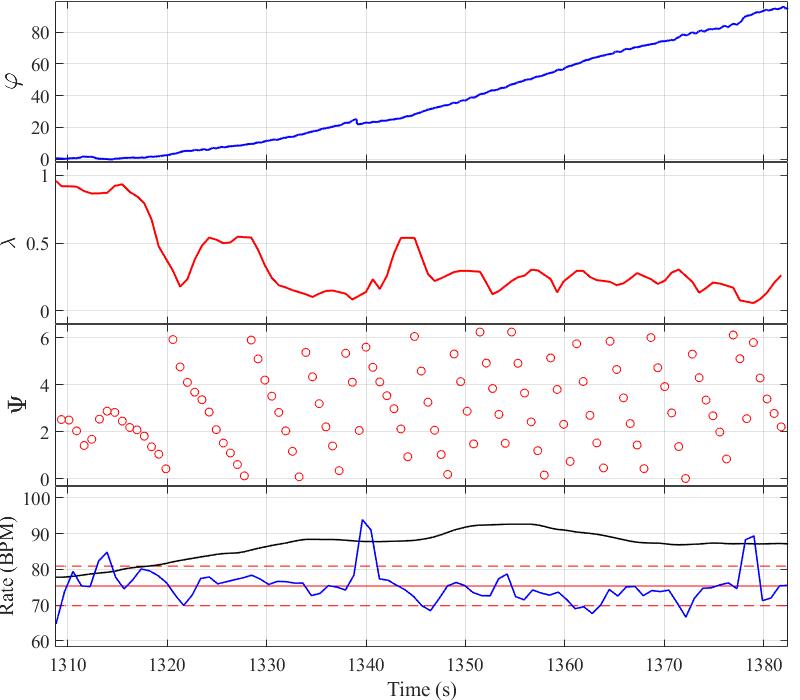 |
| 5 | 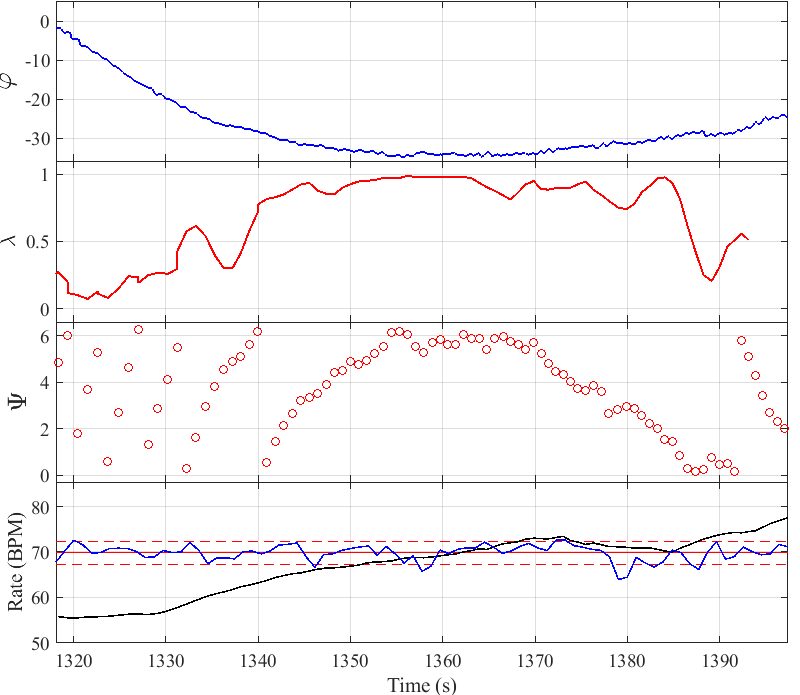 | 6 | 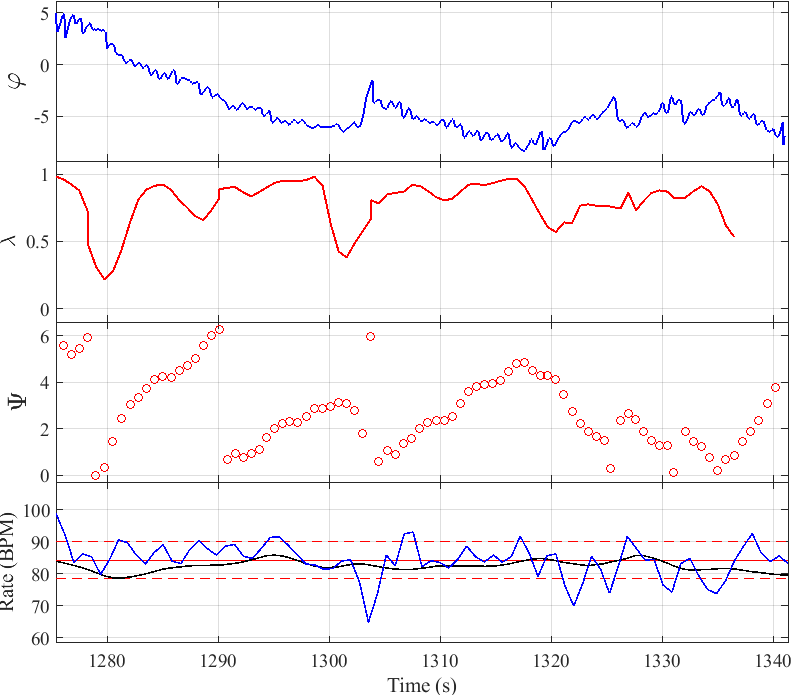 |
| 7 | 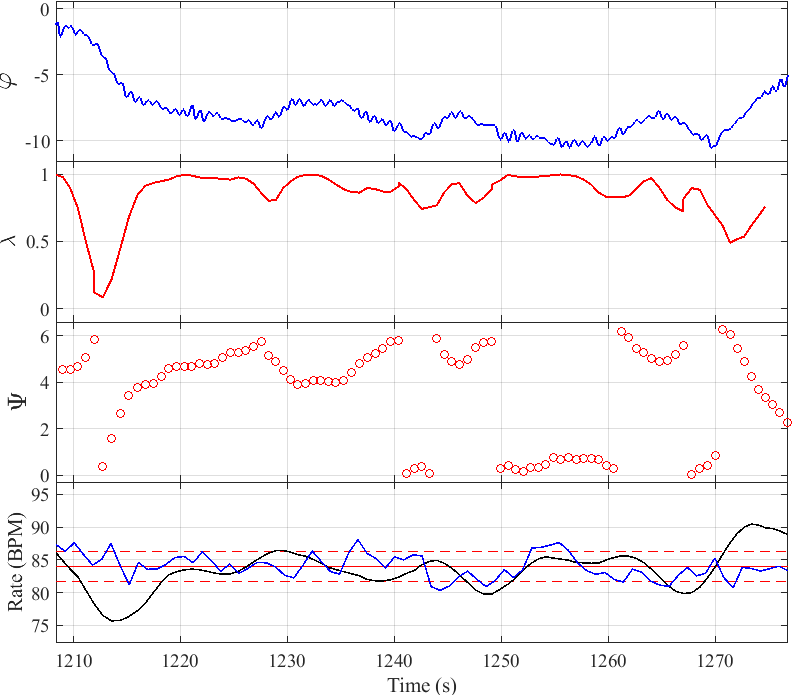 | 8 | 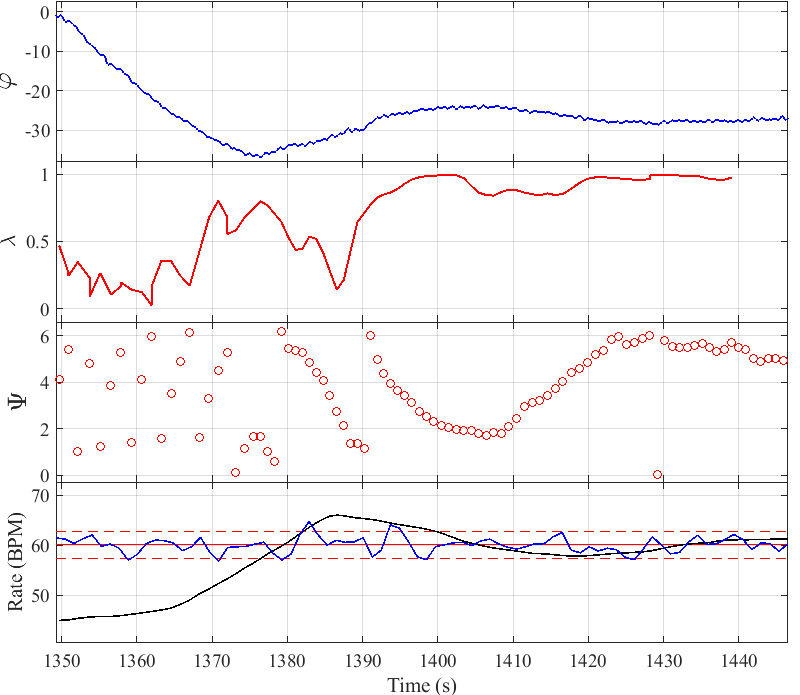 |
| 9 | 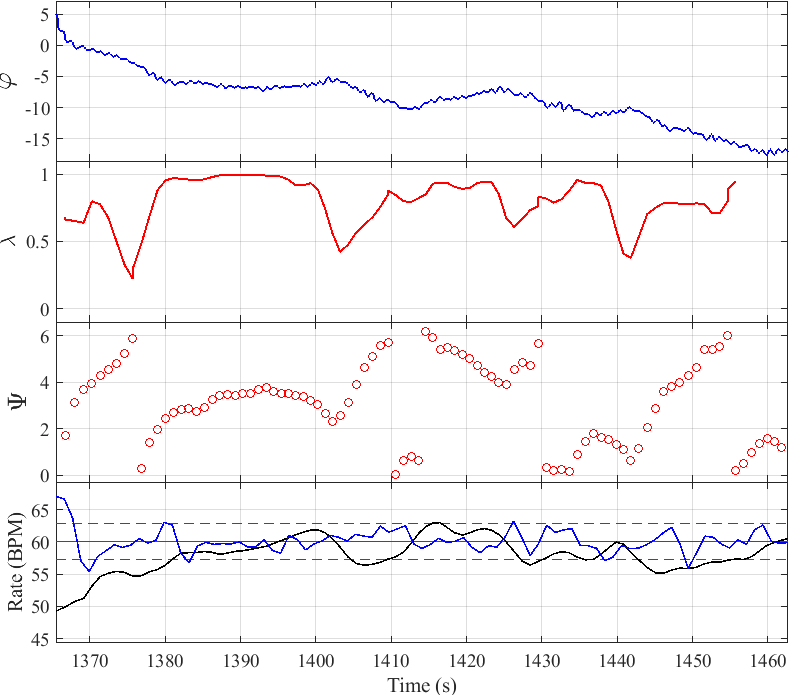 | 10 | 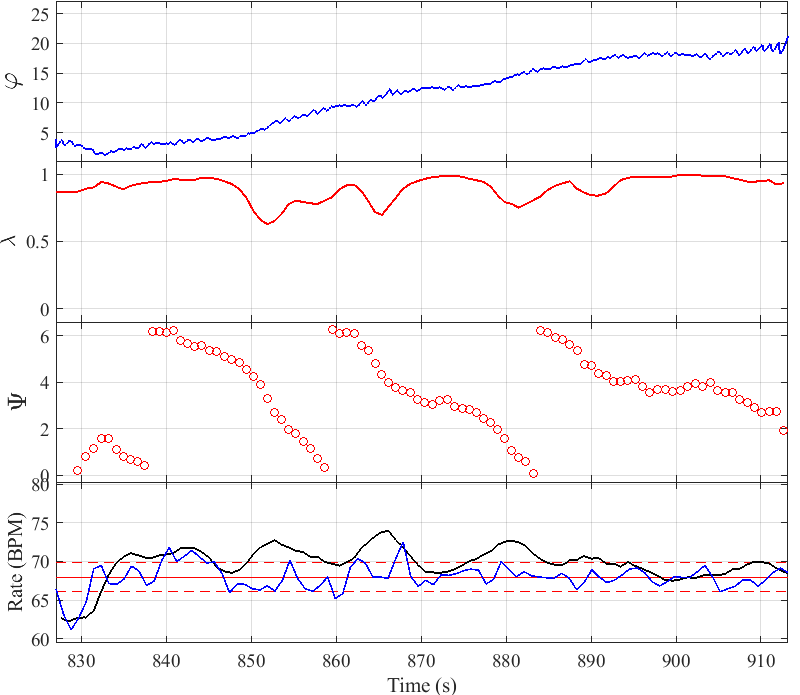 |
| 11 | 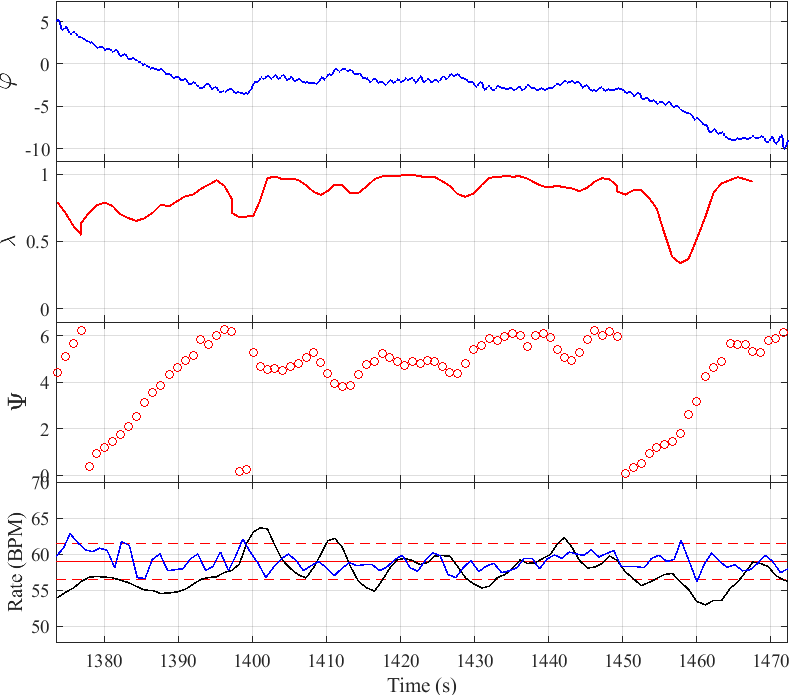 | 12 | 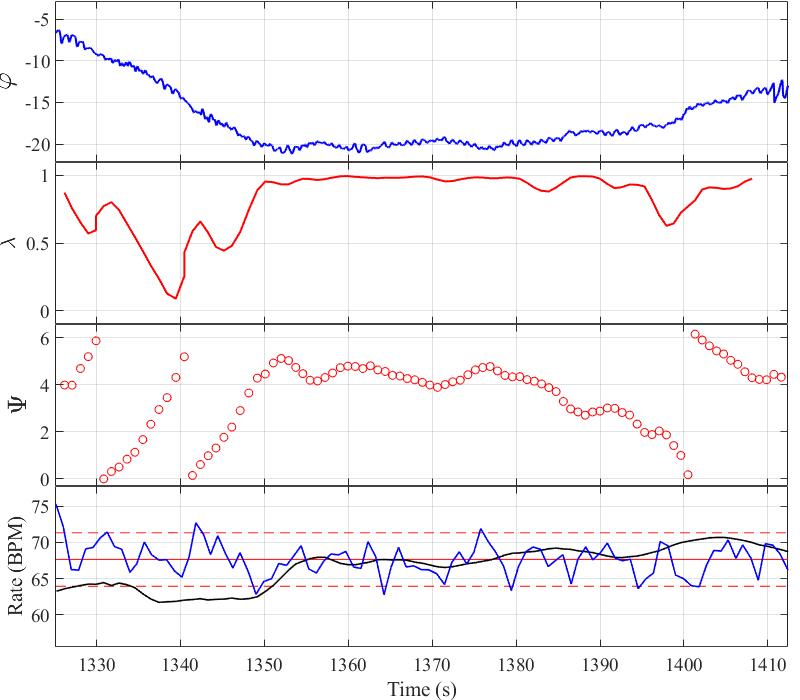 |
| 13 | 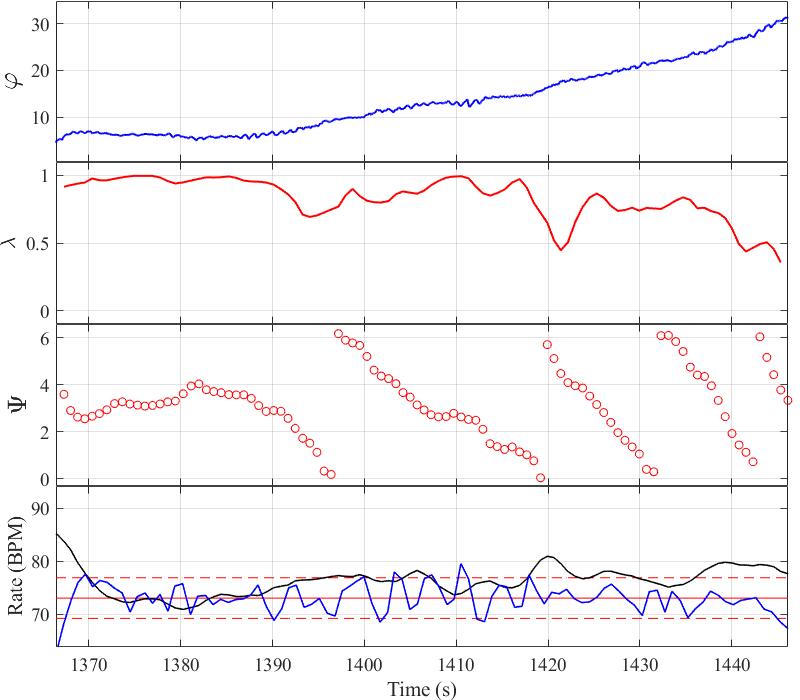 | 14 | 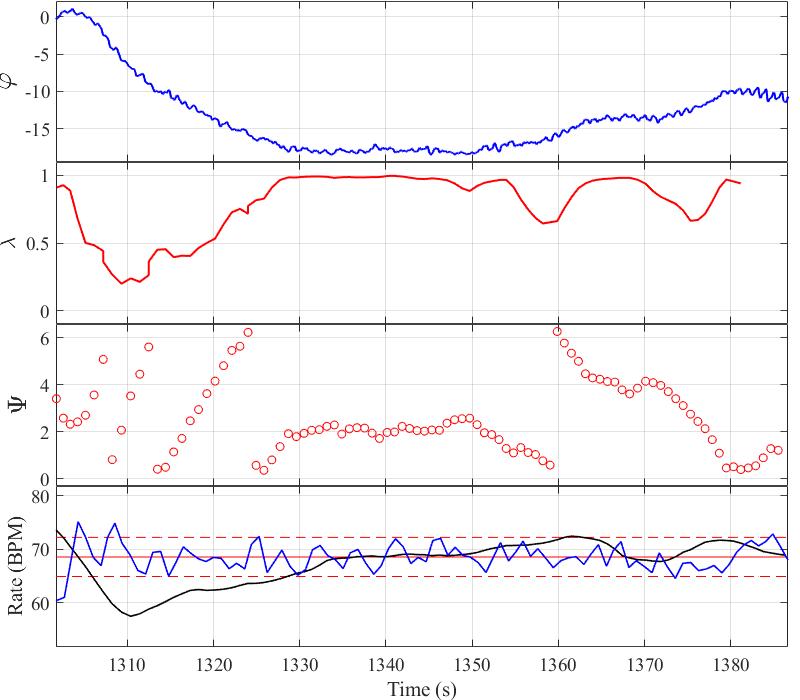 |
| 15 | 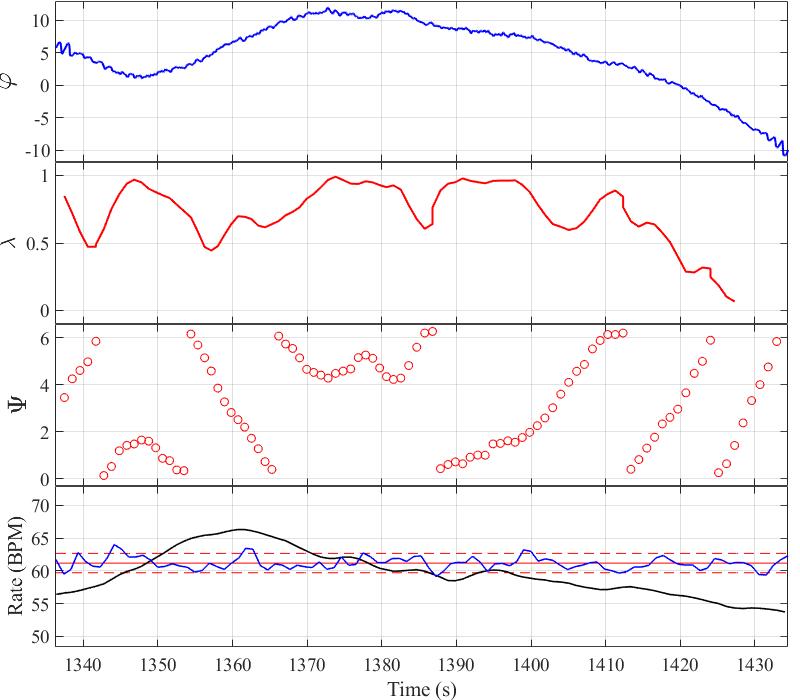 | 16 | 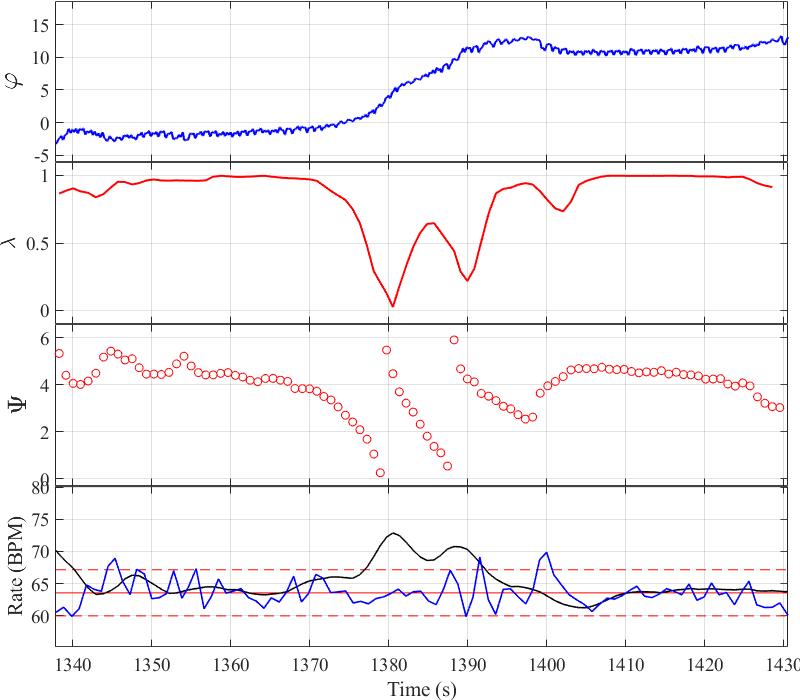 |
| 17 | 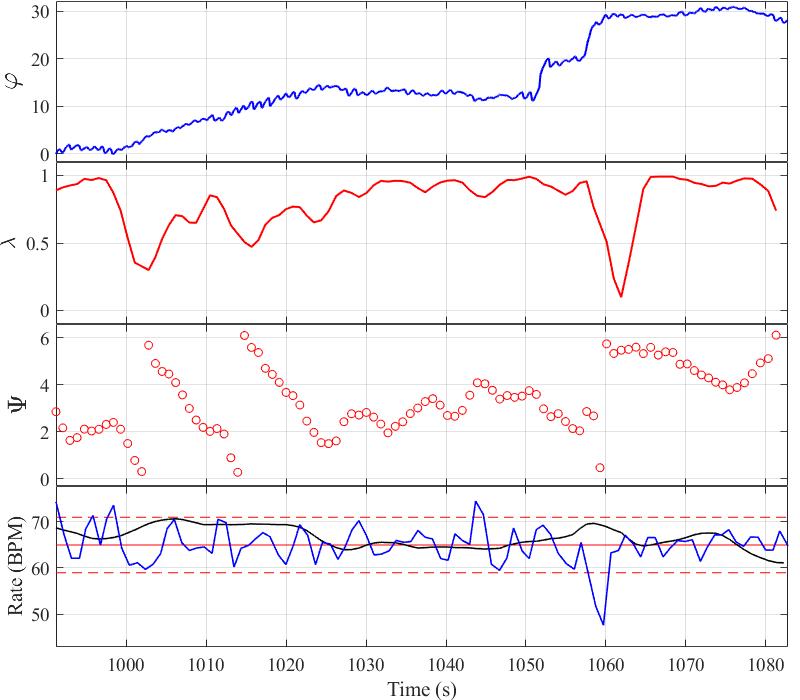 | 18 | 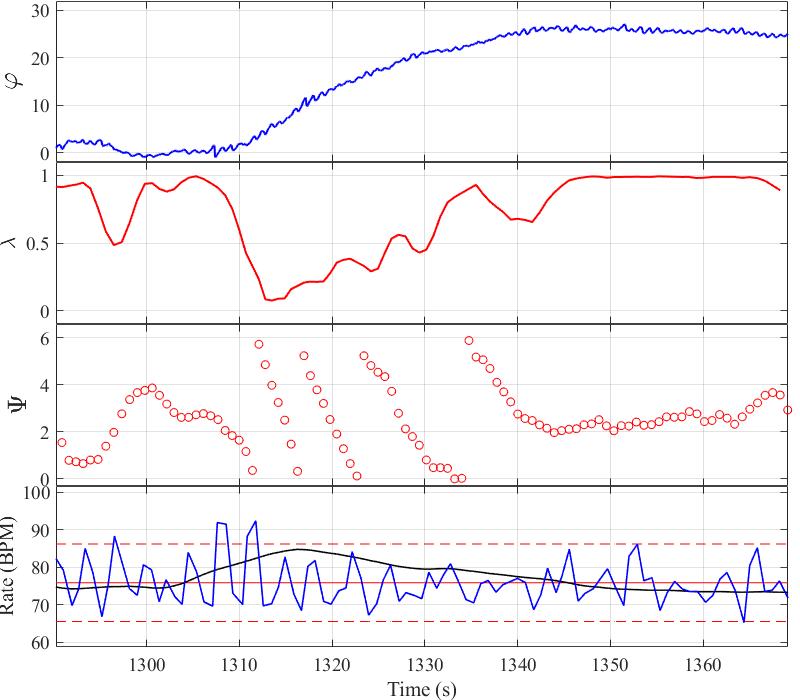 |
| 19 | 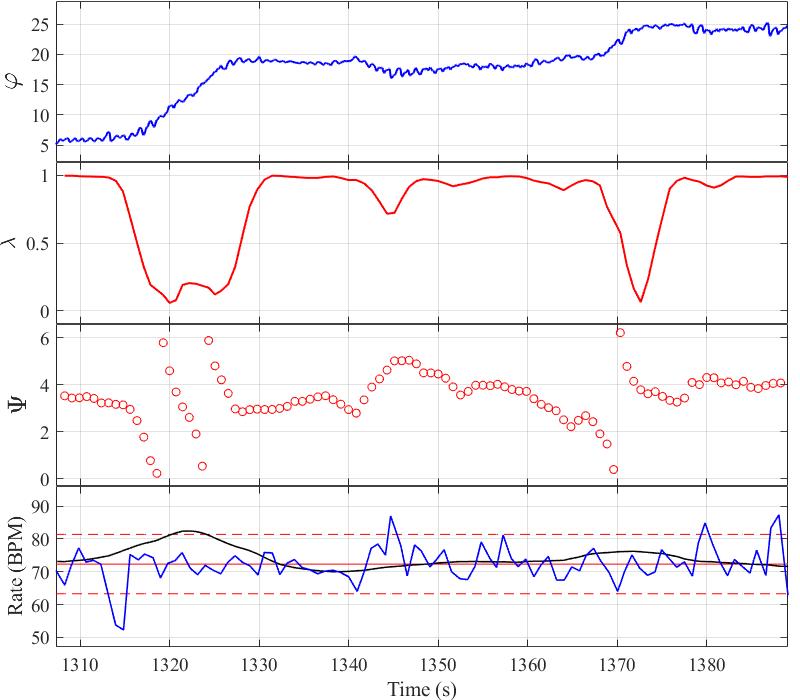 | 20 | 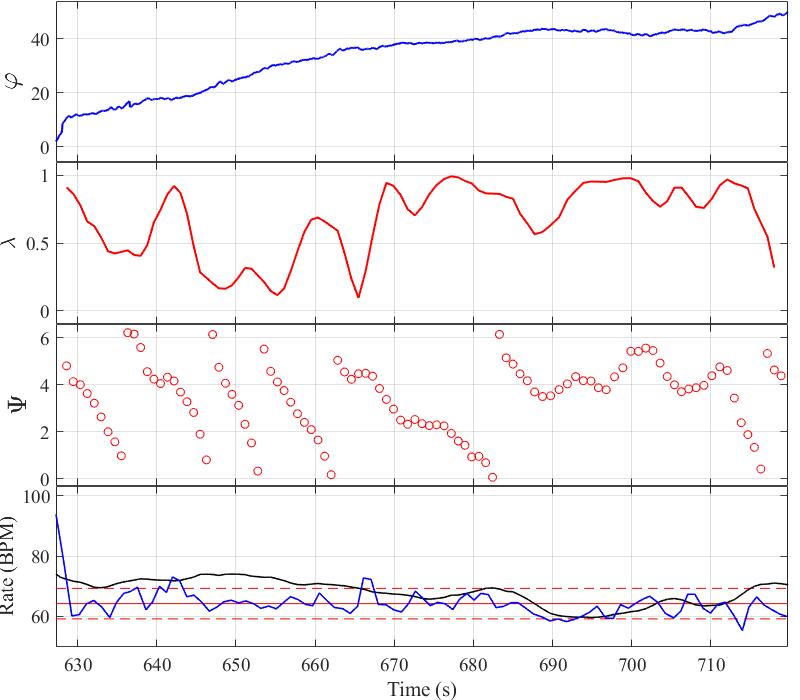 |
| 21 | 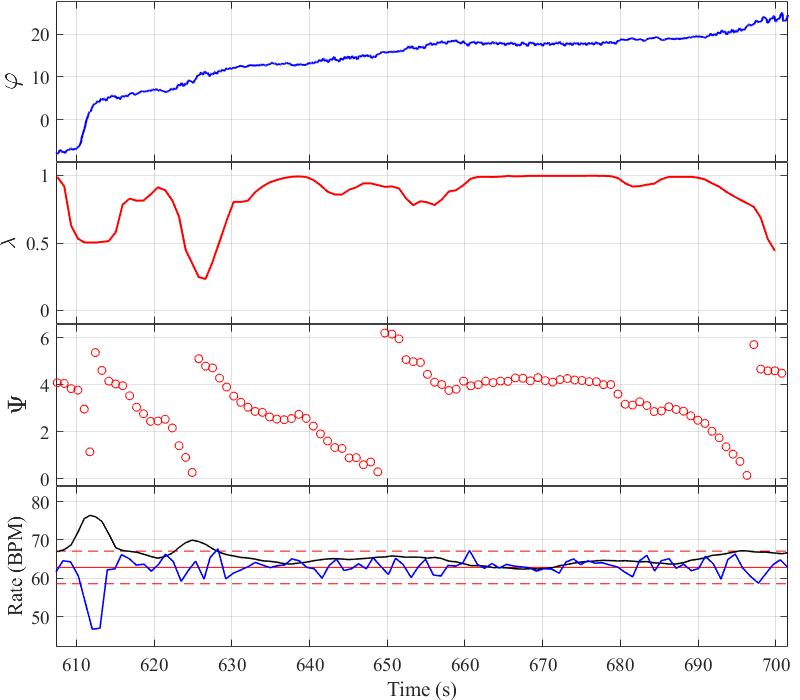 | 22 | 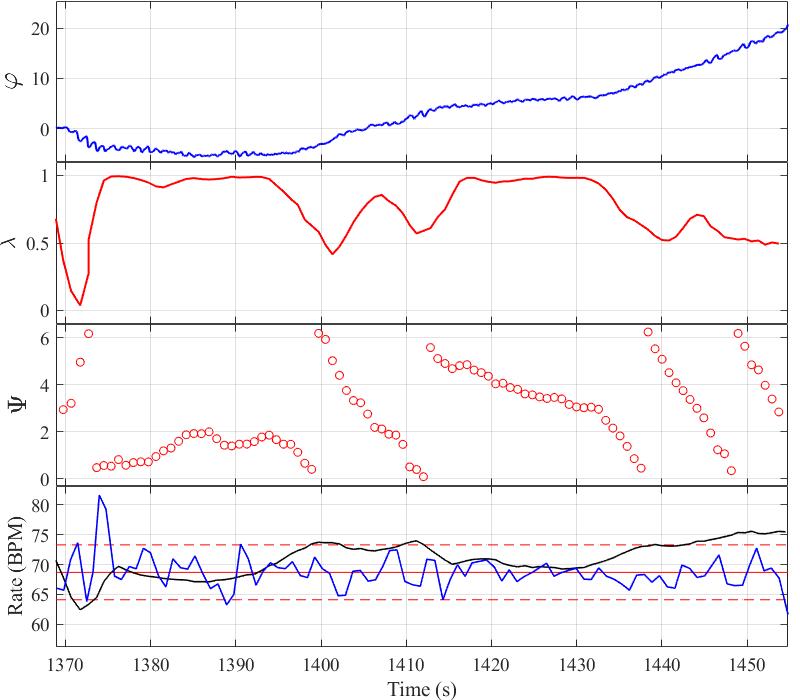 |

Figure SI3. Synchronization measures for each volunteer for the interval where phase synchronization was observed. Numbers to the left correspond to each volunteer’s numeration. Four panels for each interval show (from top to bottom): phase difference (first panel), where a limited, or close to constant value for an extended period is indicative of coupling between the two signals, as their rates become equivalent; synchronization index (second panel), where a value close to one represents 1:1 synchronization between two oscillating signals; synchrogram (third panel), with wrapped phase difference, areas limited by 2*π* can be identified, representing a particular limited phase difference between two signals; and averaged heart (black line) and respiratory (blue line) rates (fourth panel) illustrate the presence of a strong stochastic component in both heart and breathing rates. In the fourth panel red lines specify mean value (solid red line) and standard deviation (dashed red lines) of breathing rate for each interval. During episodes of synchronization the black line in the fourth panel is expected to fall wholly between the dashed red lines. A comparison of the panels shows good correspondence between different characteristics of phase synchronization: close to one synchronization index corresponds to a plateau in synchrogram and phase difference.
